# IRES-mediated Wnt2 translation in apoptotic neurons triggers astrocyte dedifferentiation

**DOI:** 10.1038/s41536-022-00248-1

**Published:** 2022-09-02

**Authors:** Hong Fan, Jialei Yang, Kun Zhang, Junling Xing, Baolin Guo, Honghui Mao, Wenting Wang, Yingzhou Hu, Wei Lin, Ying Huang, Jian Ding, Caiyong Yu, Fanfan Fu, Li Sun, Jing Wu, Youyi Zhao, Wenbin Deng, Chengji Zhou, Mengsheng Qiu, Shengxi Wu, Yu-Qiang Ding, Yazhou Wang

**Affiliations:** 1 Department of Neurobiology and Institute of Neurosciences, School of Basic Medicine, Fourth Military Medical University, 169 Chang Le Xi Road, Xi’an, Shaanxi 710032 China; 2 Department of Neurology, The Second Affiliated Hospital of Xi’an Jiaotong University, Xi’an, Shaanxi 710004 China; 3 China National Clinical Research Center for Neurological Diseases, Department of Neurology, Beijing Tiantan Hospital, No.119 Southwest of the Fourth Ring Road, 100070 Beijing, China; 4 Department of Radiation Biology, Faculty of Preventive Medicine, Fourth Military Medical University, Xi’an, China; 5 Key Laboratory of Animal Models and Human Disease Mechanisms of Chinese Academy of Sciences & Yunnan Province, Kunming Institute of Zoology, Chinese Academy of Sciences, Kunming, Yunnan 650223 China; 6 Department of Neurosurgery, Xijing Hospital, Fourth Military Medical University, 127 Chang Le Xi Road, Xi’an, Shaanxi 710032 China; 7 State Key Laboratory of Medical Neurobiology and MOE Frontiers Center for Brain Science, Institute of Brain Science, Fudan University, Shanghai, 200032 China; 8 Center for Mitochondrial Biology and Medicine, The Key Laboratory of Biomedical Information Engineering of Ministry of Education, School of Life Science and Technology, Xi’an Jiaotong University, 28 Xian Ning Xi Road, Xi’an, China; 9 School of Pharmaceutical Sciences (Shenzhen), Sun Yat-sen University and Jiangxi Deshang Pharmaceutical Co., Ltd., Zhangshu, 336000 China; 10 Institute for Pediatric Regenerative Medicine of Shriners Hospitals for Children, Department of Biochemistry and Molecular Medicine, University of California at Davis, School of Medicine, 2425 Stockton Blvd, Sacramento, CA 95817 USA; 11 Institute of Developmental and Regenerative Biology, Key Laboratory of Organ Development and Regeneration of Zhejiang Province, College of Life Sciences, Hangzhou Normal University, Hangzhou, 310029 China

**Keywords:** Stroke, Adult neurogenesis

## Abstract

Reactive astrogliosis usually bears some properties of neural progenitors. How injury triggers astrocyte dedifferentiation remains largely unclear. Here, we report that ischemia induces rapid up-regulation of Wnt2 protein in apoptotic neurons and activation of canonical Wnt signaling in reactive astrocytes in mice, primates and human. Local delivery of Wnt2 shRNA abolished the dedifferentiation of astrocytes while over-expressing Wnt2 promoted progenitor marker expression and neurogenesis. Both the activation of Wnt signaling and dedifferentiation of astrocytes was compromised in ischemic caspase-3^−/−^ cortex. Over-expressing stabilized β-catenin not only facilitated neurogenesis but also promoted functional recovery in ischemic caspase-3^−/−^ mice. Further analysis showed that apoptotic neurons up-regulated Wnt2 protein via internal ribosome entry site (IRES)-mediated translation. Knocking down death associated protein 5 (DAP5), a key protein in IRES-mediated protein translation, significantly diminished Wnt activation and astrocyte dedifferentiation. Our data demonstrated an apoptosis-initiated Wnt-activating mechanism which triggers astrocytic dedifferentiation and facilitates neuronal regeneration.

## Introduction

Reactive astrogliosis is a hallmark pathologic change of cerebral ischemia and various neurological diseases. Upon ischemia, astrocytes start to proliferate and become morphologically hypertrophic. It has now been accepted that reactive astrocytes are heterogeneous and play diverse roles after injury^1^. The phenotype and function of reactive astrocytes depend on the dynamics of different sub-populations of reactive astrocytes^2^. Recent progresses revealed that reactive astrocytes could promote rather than inhibit axon growth as early studies had thought^3–5^. Therefore, elucidating the mechanism underlying the phenotype changes of reactive astrocytes is important and valuable.

One outstanding feature of reactive astrocytes is that some reactive astrocytes up-regulate many genes associated with neural progenitors and can form neurospheres in vitro^6,7^, indicating certain degree of dedifferentiation. Because adult neural stem cells (NSCs) share many common properties with astrocytes, it has been hypothesized that reactive astrocytes may undergo some degree of dedifferentiation^8–10^. Elucidating how reactive astrocytes acquire the dedifferentiated phenotype, particularly the possible neurogenic potential, is important for understanding how central nerve system (CNS) responds to injury and will shed light on facilitating repair/regeneration of CNS.

Some studies have attempted to disclose the mechanisms relating to this phenotypic change of reactive astrocytes. In striatum, Notch signaling which normally maintains the quiescence of adult NSCs, is inhibited after ischemia^11^. This liberates the neurogenic potential of local astrocytes^11^. In the cerebral cortex, Shh signaling is up-regulated quickly following ischemia and has been demonstrated to be required for the in vitro neurosphere formation of reactive astrocytes^12^. Activating Shh signaling stimulated proliferation of reactive astrocytes in vivo^12^. Other studies focused on the neuronal reprogramming potential of astrocytes by artificially manipulating key proteins involved in neuronal differentiation, such as Sox2, PTB1, Ngn2 and NeuroD1^13–17^, although some of these in vivo reprogramming was recently challenged^18^. These studies revealed the cell fate plasticity of astrocytes. However, under ischemia, how local environment triggers the appearance of progenitor properties of reactive astrocytes, particularly in vivo, remains still unclear.

In the present study, we focused on Wnt/β-catenin signaling, an important niche signaling in maintaining the active status of adult NSCs and supports neurogenesis in the subventricular zone (SVZ) and hippocampus^19,20^, and explored the response of Wnt/β-catenin signaling to ischemia and its roles in the dedifferentiation of astrocytes and neurogenesis in cerebral cortex. Our data surprisingly revealed that apoptotic neurons up-regulate Wnt2 protein via internal ribosome entry site (IRES) mediated protein translation, which induces the dedifferentiation response of reactive astrocytes and supports cortical neurogenesis.

## Results

### Focal ischemia induces Wnt signaling activation and dedifferentiation of reactive astrocytes

**Fig. 1:**
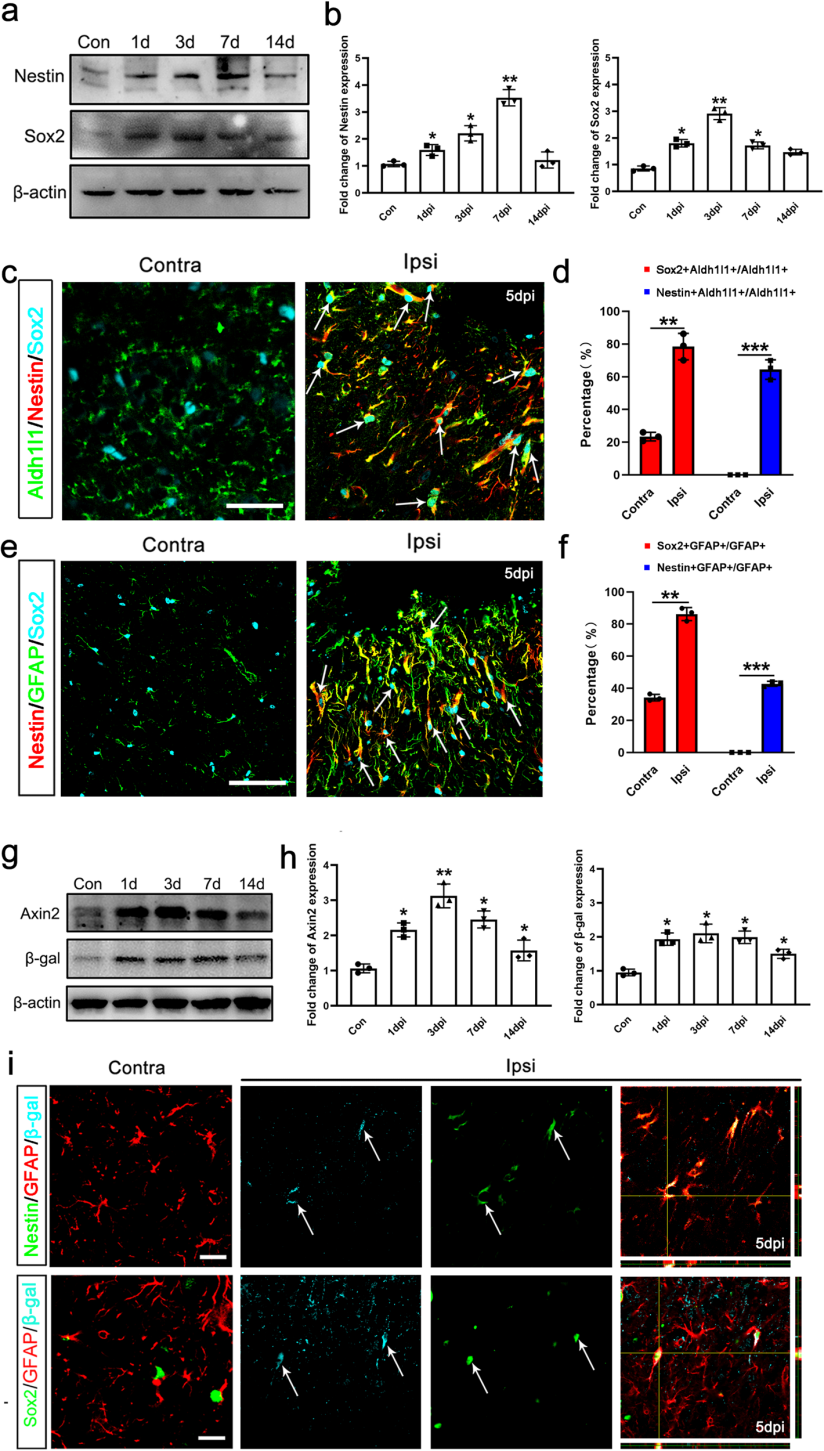
Dedifferentiation and activation of Wnt/β-catenin signaling in reactive astrocytes after cerebral ischemia. **a**, **b** Western-blotting and quantification of Nestin and Sox2 in ischemic cortex at different time points. *P_Nestin-1dpi_ = 0.031, *P_Nestin-3dpi_ = 0.033, **P_Nestin-7dpi_ = 0.0026, *P_Sox2-1dpi_ = 0.029, **P_Sox2-3dpi_ = 0.0031, *P_Sox2-7dpi_ = 0.028. One-way ANOVA followed by Bonferroni’s post hoc comparisons tests. *N* = 3 mice per time points. **c** Triple-immunostaining of Aldh1l1/Nestin/Sox2 in contralateral (Contra) and ipsilateral (Ipsi) side of ischemic cortex at 5 dpi. Bars = 50 μm. **d** Quantification of Sox2/Aldh1l1- and Nestin/Aldh1l1-positive cells in the zone expanded 200 μm from lesion border. *N* = 3 mice per group. **e** Triple-immunostaining of Nestin/GFAP/Sox2. Bar = 50 μm. **f** Quantification of Sox2/GFAP-positive cells and Nestin/GFAP-positive cells in the zone expanded 200 μm from lesion border. **g**, **h** Western-blotting and quantification of Axin2 and β-gal in ischemic cortex at different time points post injury. Notice the up-regulation of Axin2 and β-gal from 1 dpi. *P_Axin2-1dpi_ = 0.019, **P_Axin2-3dpi_ = 0.0024, *P_Axin2-7dpi_ = 0.012, *P_Axin2-14dpi_ = 0.035, *P_βgal-1dpi_ = 0.017, *P_βgal-3dpi_ = 0.021, *P_βgal-7dpi_ = 0.018, *P_βgal-14dpi_ = 0.027. One-way ANOVA followed by Bonferroni’s post hoc comparisons tests. *N* = 3 mice per time points. **i** Representative images of triple-immunostaining of Nestin/GFAP/β-gal and Sox2/GFAP/β-gal in Topgal mice at 5 dpi. Bar = 10 μm. Notice the induction of β-gal in Nestin- and Sox2-positive astrocytes in the injured cortex (Ipsi). Arrowheads point to triple-positive cells in (**c**, **e**, **i**). Mean ± standard error (SE). Asterisks indicate the comparison with control group. Asterisks with bars connecting two groups indicate difference between these two groups.

To probe the local signals that trigger the phenotype change of astrocytes, we adopted a photothrombosis focal ischemia model. Neural progenitor markers, Nestin and Sox2 were quickly up-regulated in the injury site from one day post ischemia (Fig. 1a and b). Both Nestin and Sox2 were mainly expressed by astrocytes, as showed by immunostaining of Aldh1l1 and GFAP (Fig. 1c–f). Only a minor portion of Nestin and Sox2-positive cells expressed oligodendrocyte precursor marker NG2 or microglia marker Iba-1 (Supplementary Fig. 1a–d). BrdU-labeling assay showed that, there was no BrdU-positive cell in the normal cortex (data now shown) while approximately one third of astrocytes around the lesion border were labeled by BrdU (Supplementary Fig. 1e, f). Most of these Brdu-labeled astrocytes were either Nestin- or Sox2-positive (Supplementary Fig. 1e, f). As SVZ progenitors can contribute to the reactive astrocytes around the infarction core^21,22^, we tested if local astrocytes could change their phenotype. We injected Tamoxifen (TAM) to Nestin-CreER:ROSA-DTA mice for 3 days before ischemia to deplete SVZ neural progenitors and examined Nestin expression at 7 days post injury (dpi). The results showed that TAM effectively ablated Nestin-positive progenitors in SVZ. However, large number of Nestin/GFAP-positive cells were still detected in the lesion cortex (Supplementary Fig. 2). Taken together, these data demonstrated that reactive astrocytes in the ischemic cortex undergo certain degree of dedifferentiation upon injury.

To explore the possible mechanism of this injury-induced dedifferentiation, we focused on canonical Wnt signaling because it plays essential roles in the self-renewal of neural stem cells (NSCs) during development and in the neurogenesis of adult NSCs in subventricular zone and hippocampus^19^. To directly readout if canonical Wnt signaling were re-activated in reactive astrocytes, we adopted a widely-used Wnt signaling reporter mouse line, Topgal mouse^23^. There was no or very low expression of Wnt signaling reporter β-gal and Wnt signaling target gene Axin2 in intact cortex. After injury, β-gal and Axin2 was up-regulated from 1 dpi (Fig. 1g, h), similar to the time course of Nestin and Sox2 up-regulation. To dissect the cell types in which Wnt signaling was activated, we performed triple-immunostaining of β-gal/GFAP/Nestin and β-gal/GFAP/Sox2. There were no β-gal-positive cells in the control side of cortex. In the ischemic cortex, β-gal expression was induced mainly in Nestin/GFAP- or Sox2/GFAP-positive reactive astrocytes (Fig. 1i). These data suggested that canonical Wnt signaling is activated in reactive astrocytes and in coincidence with the expression of progenitor markers in the ischemic cortex.

### Apoptotic neurons up-regulate Wnt2 protein which activates Wnt signaling

**Fig. 2:**
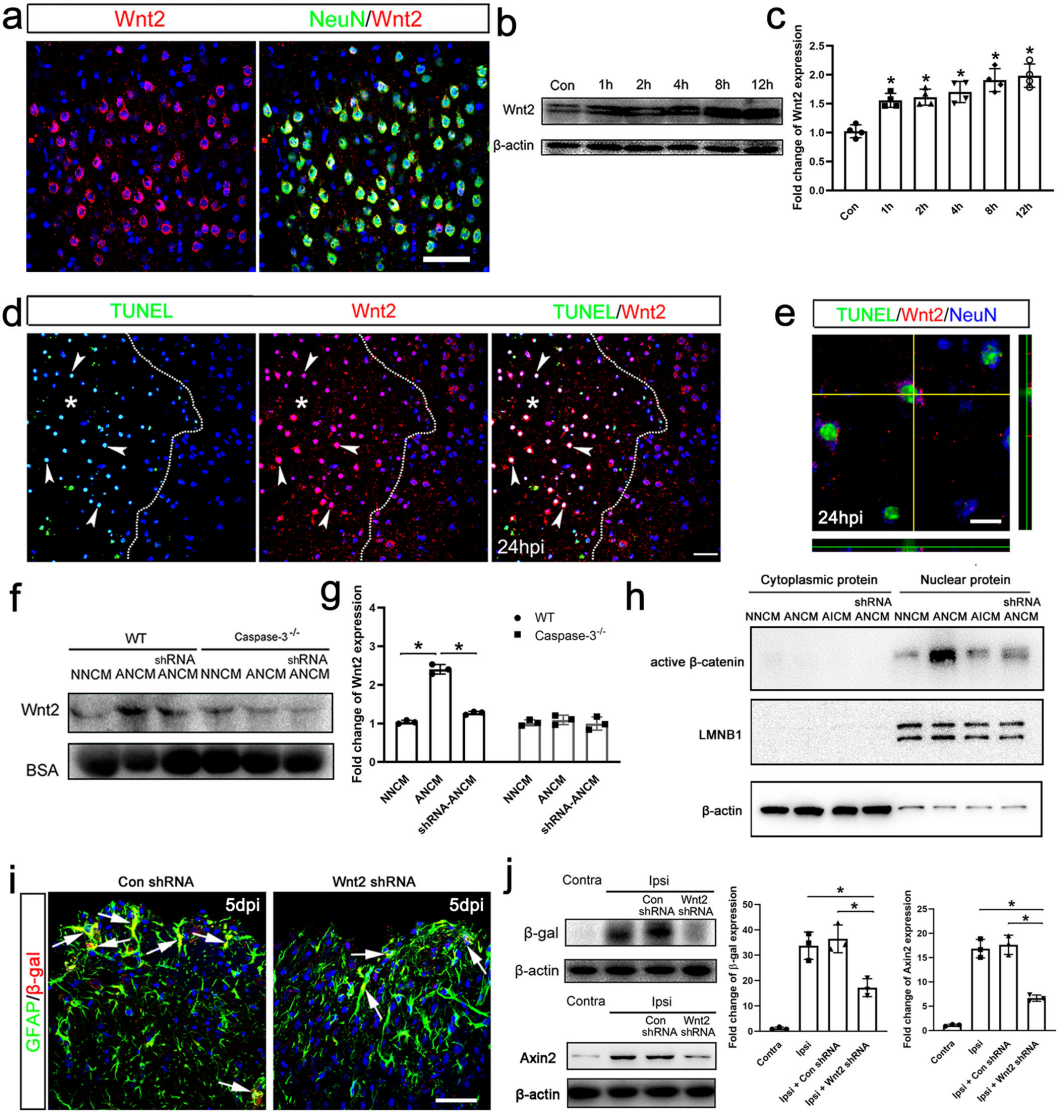
Up-regulation of Wnt2 by apoptotic neurons and its involvement in Wnt//β-catenin signaling activation. **a** Double-immunostaining of NeuN/Wnt2 in normal cortex. Bar = 50 μm. **b**, **c** Western-blotting of Wnt2 at different time points in ischemic cortex. Notice that Wnt2 protein increases quickly after ischemia. *P = 0.026 (1 h), 0.028 (2 h), 0.024 (4 h), 0.048 (8 h), 0.043 (12 h). One-way ANOVA followed by Bonferroni’s post hoc comparisons tests. *N* = 4 mice per group. **d** Combination of TUNEL staining and Wnt2 immunostaining at 24 hpi. Wnt2^high^ immunoreactivity overlapped well with TUNEL-staining (arrowheads). Dashed line indicates lesion border and asterisk indicates lesion area. Bar = 30 μm. **e** Representative image of TUNEL/Wnt2/NeuN-triple immunostaining in the lesion area. Bar = 10 μm. **f**, **g** Western-blotting of Wnt2 in conditioned medium of normal cultured (NNCM), apoptotic (ANCM), Wnt2 shRNA pretreated apoptotic (shRNA ANCM) wild type and caspase-3^−/−^ neurons. Apoptotic neurons increase Wnt2 release into the culture medium, and this increase is compromised by caspase-3 ablation and Wnt2 shRNA. BSA was used as internal control. *P_NNCM vs ANCM_ = 0.012, *P_ANCM vs ShRNA-ANCM_ = 0.014. One-way ANOVA followed by Bonferroni’s post hoc comparisons tests. *N* = 3 batches of cells. **h** Western-blotting of active β-catenin in the cytoplasmic and nuclear protein of astrocytes treated by NNCM, ANCM, AICM or shRNA ANCM. ANCM greatly increases the level of β-catenin in nuclear protein. LMNB1 was used as nuclear protein control. **i** Double-immunostaining of GFAP/β-gal in Topgal mice pretreated with Wnt2 shRNA. Arrows point to representative double-positive cells. Bar = 50 μm. **j** Western-blotting of β-gal and Axin2 in ischemic cortex of Topgal mice treated with Wnt2 shRNA or control. Left: *P_ipsi vs ipsi+Wnt2-shRNA_ = 0.038, *P_ipsi+Con-shRNA vs ipsi+Wnt2-shRNA_ = 0.041, Right: *P_ipsi vs ipsi+Wnt2-shRNA_ = 0.013, *P_ipsi+Con-shRNA vs ipsi+Wnt2-shRNA_ = 0.016. One-way ANOVA followed by Bonferroni’s post hoc comparisons tests. *N* = 3 mice per group. Notice the less induction of β-gal and Axin2 in Wnt2 shRNA pretreated cortex. Mean ± standard error (SE). Asterisks indicate the comparison with control group. Asterisks with bars connecting two groups indicate difference between these two groups.

To probe the mechanism of Wnt activation, we first examined the expression of Wnt ligands in cortex by real time RT-PCR. Among the 18 Wnts, Wnt2, Wnt4, Wnt7a, Wnt9a, and Wnt10a showed a relatively high expression level in the intact cortex, as compared with house-keeping gene *Gapdh* in the same tissue (Supplementary Fig. 3a). Because Wnt4 acts as a non-canonical Wnt ligand in the CNS^24,25^, we performed in situ hybridization of *Wnt2, Wnt7a, Wnt9a* and *Wnt10a*. Among these, *Wnt2* mRNA was expressed in abundance by more cells (Suppl. Fig. 3b). To identify the cell type which expresses Wnt2, we performed immunohistochemistry. Double staining of Wnt2 with NeuN or GFAP showed that Wnt2 is mainly expressed by neurons (Fig. 2a, Supplementary Fig. 3c). Western-blotting of cortical tissue showed a higher molecular weight of Wnt2 than predicted, possibly due to posttranslational modification. To confirm the specificity of Wnt2 antibody used, we injected cortex with lentivirus expressing Wnt2 shRNA, which significantly reduced the level of Wnt2 mRNA (Supplementary Fig. 3d) as well as Wnt2 protein (Supplementary Fig. 3e).

After ischemia, the levels of Wnt2 protein increased quickly in comparison with that in intact cortex (Fig. 2b, c). No significant changes of Wnt4, Wnt7a, Wnt9a and Wnt10a protein were observed (Supplementary Fig. 3f–i). Interestingly, most of the cells with high level of Wnt2 immunoreactivity (Wnt2^hi^) in the injury site were TUNEL positive (Fig. 2d), exhibiting a nuclear-like staining owning to the shrinkage of cytoplasm during apoptosis. Triple-immunostaining showed that TUNEL/Wnt2-positive immunoreactivity overlapped well with NeuN-immunoreactivity (Fig. 2e), indicating the up-regulation of Wnt2 by apoptotic neurons. To confirm whether apoptotic neurons up-regulated Wnt2, we cultured primary neurons and treated neurons by oxygen-glucose deprivation (OGD) to mimic ischemia. Starting from 2 h post OGD treatment, the levels of Wnt2 and cleaved caspase-3 (CC3) increased simultaneously in cultured neurons (Supplementary Fig. 3j). Adding pan-caspase inhibitor z-VAD effectively blocked the up-regulation of Wnt2 in apoptotic neurons, as compared with control cells (Supplementary Fig. 3j). Same OGD condition did not activate Caspase-3 in astrocyte culture (Supplementary Fig. 3k). To investigate whether Wnt2 was released from apoptotic neurons, we analyzed the levels of Wnt2 protein in conditioned medium (CM) from normally-cultured neurons (NNCM), apoptotic neurons (ANCM) prepared with OGD treatment, and apoptotic neurons treated with Wnt2 shRNA (shRNA ANCM). ELISA showed significant increase of Wnt2 levels in OGD-treated cells, while this increase was compromised by Wnt2 shRNA treatment (Suppl. Fig. 3l). Western-blotting confirmed that OGD induced notable large amount of Wnt2 in the ANCM of wild type (WT) neurons, but not in the ANCM of *Capase-3* deficient neurons (Fig. 2f, g). This apoptosis-induced Wnt2 protein could be effectively blocked by pretreating neurons with Wnt2 shRNA (shRNA-ANCM) which did not affect the expression of caspase-3 by itself (Fig. 2f, g, and Supplementary Fig. 3m). These data demonstrated that apoptotic neurons up-regulate and release Wnt2.

To investigate whether the released Wnt2 was sufficient to activate Wnt/β-catenin signaling, we stimulated primary astrocytes with NNCM, ANCM, shRNA ANCM or CM from apoptosis-inhibited neurons (AICM). The results showed that ANCM dramatically increased the amount of active β-catenin (unphosphorylated β-catenin) in the cell nucleus in relative to NNCM, AICM, and shRNA ANCM (Fig. 2h), suggesting that Wnt2 released by apoptotic neurons was able to activate Wnt/β-catenin signaling in astrocytes in vitro. Further, we injected lentivirus expressing Wnt2 shRNA into the cerebral cortex 9 days before ischemia and examined Wnt activation at 5 dpi. To evaluate the cell types infected, lentivirus expressing GFP was injected in parallel and GFP expression was examined before ischemia. Approximately 61.13 ± 4.62% of GFP-labeled cells were NeuN-positive, 29.43 ± 3.92% GFAP-positive, 5.37 ± 1.07% NG2-positive, and 4.07 ± 0.55% Iba-1-positive (Suppl. Fig. 4). Both immunohistochemistry and Western-blotting revealed that the induction of Wnt reporter β-gal and Wnt target Axin2 was remarkably attenuated in Wnt2 shRNA pre-treated cortex (Fig. 2i, j). These data indicated that injury-induced Wnt2, particularly from apoptotic neurons, activates Wnt signaling in the reactive astrocytes.

### Wnt2 is required for astrocyte dedifferentiation and facilitates neurogenesis

**Fig. 3:**
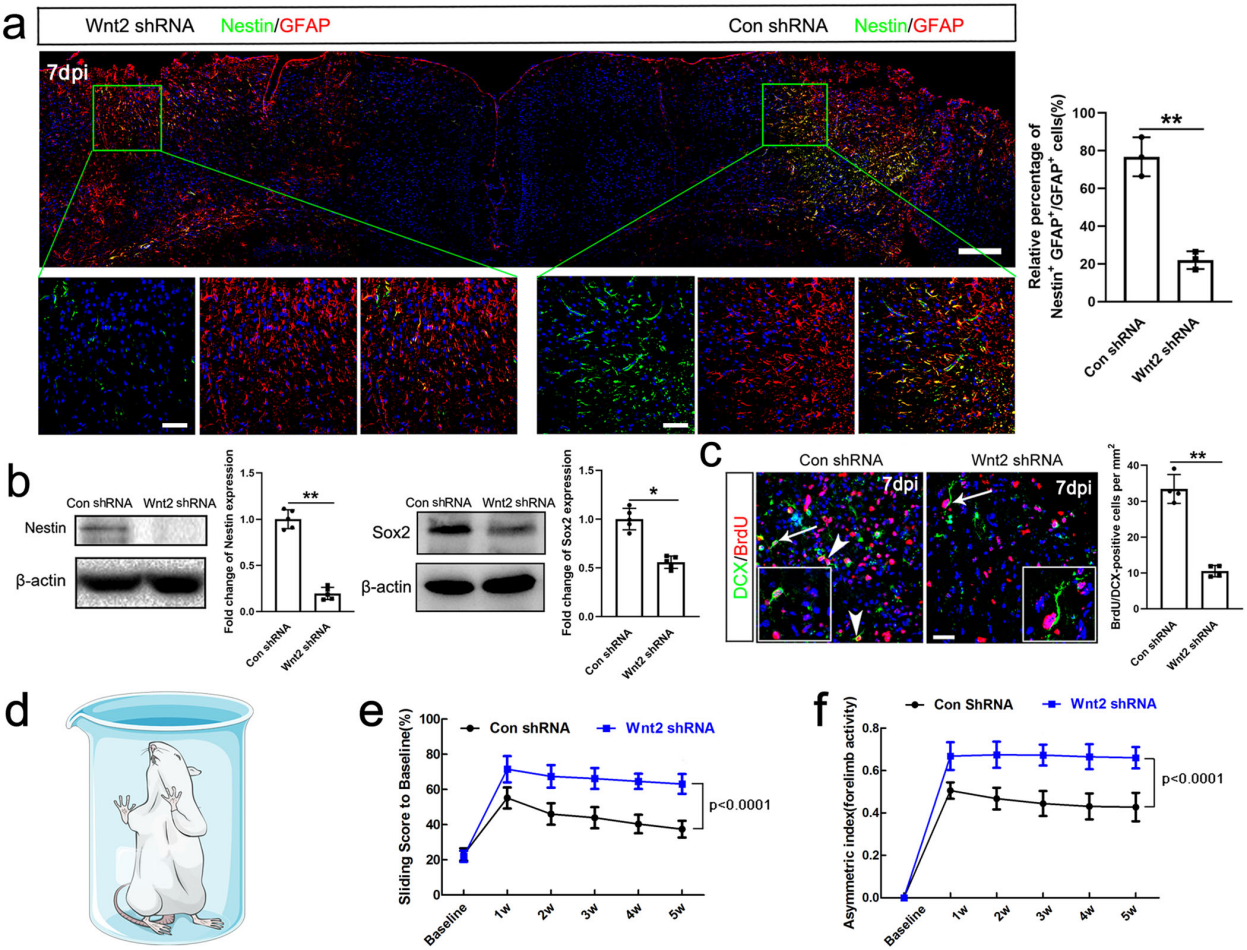
Effects of Wnt2 knockdown on astrocyte dedifferentiation and cortical neurogenesis. **a** Representative images of Nestin/GFAP double-immunostaining in the bilateral cortice with two ischemic injures infected by lentivirus expressing control shRNA (right) and Wnt2 shRNA (left), respectively. Bars = 200 μm in upper panel. Bar = 100 μm in enlarged view. **b** Western-blotting of Nestin and Sox2 in Wnt2 shRNA treated mice at 7 dpi. Wnt2 shRNA dramatically suppressed the expression of Nestin and Sox2 without obviously affecting the expression of GFAP. *N* = 5 mice per group. **c** Double-immunostaining and quantification of DCX/BrdU in ischemic cortices treated by control shRNA or Wnt2 shRNA. Arrowheads point to double-positive cells. Arrows point to magnified cells. Bar = 20 μm. **P = 0.0087. Two-tailed Student’s *t* test. *N* = 4 mice per group. **d**–**f** Forelimb activity assay of mice treated with control shRNA or Wnt2 shRNA. From 2 weeks on, the sliding scores and the asymmetric index of forelimb activity in Wnt2 shRNA treated group were much higher than control group, indicating worse functional recovery. *N* = 5 mice per group. *P* < 0.0001 (2w, 3w, 4w and 5w). Two-way RM ANOVA. **a**–**c**, asterisks with bars connecting two groups indicate difference between these two groups. **e**, **f**, *P* values indicate comparison of experimental group with control group of same time point. Mean ± standard error (SE).

To test whether Wnt2 was involved in the acquisition of dedifferentiated phenotype of reactive astrocytes in vivo, we made focal ischemia in bilateral cortices, injected lentivirus expressing Wnt2 shRNA in the right cortex and lentivirus expressing control shRNA in the left cortex. Immunohistochemistry showed that Wnt2 shRNA dramatically down-regulated the expression of Nestin in the injured cortex without affecting the up-regulation of GFAP (Fig. 3a). Western-blotting confirmed the reduction of Nestin and Sox2 in injured cortex pretreated by Wnt2 shRNA (Fig. 3b), suggesting the requirement of Wnt2 for the dedifferentiation phenotype of reactive astrocytes. Previous studies have reported the migration of new-born neuroblasts, although very weak, from SVZ to cortex after ischemia^26,27^. Considering that astrocytes in the SVZ play important roles for maintaining the neurogenic niche^28^, we hypothesized that these Nestin-positive reactive astrocytes might provide supporting environment for neurogenesis in ischemic cortex. We then investigated the effects of Wnt2 knockdown on the post-ischemic cortical neurogenesis. Immunohistochemistry showed that DCX/BrdU-double positive cells were significantly reduced in Wnt2 shRNA-treated cortex at 7 dpi as compared with that of control shRNA-treated cortex (Fig. 3c). To explore the possible mechanism involved in this Wnt-induced astrocyte dedifferentiation, we investigated the expression of Ngn2 and NeuroD1, which are two Wnt signaling target genes and key transcription factors in neuronal fate determination and maturation. Ngn2, but not NeuroD1, was detected in reactive astrocytes following ischemia. Wnt2 shRNA effectively abolished the expression of Ngn2 (Suppl. Fig. 5a). To evaluate the functional significance of astrocyte dedifferentiation, we analyzed forelimb activity of control shRNA treated mice and Wnt2 shRNA treated mice following focal ischemia in the forelimb sensorimotor cortex by cylinder test (Fig. 3d). From 2 weeks post injury, both the sliding scores and the asymmetry of the animals’ forelimb were significantly higher in Wnt2 shRNA treated mice, indicating that inhibiting astrocyte dedifferentiation maybe harmful for the functional recovery (Fig. 3e, f).

To assess roles of the downstream signals of Wnt2 in this process, lentivirus expressing dominant-negative TCF-4 (dnTCF-4) was injected to cortex 9 days before ischemia. DnTCF-4 inhibits the activity of Wnt signaling by competing the interaction between β-catenin and TCF^29^. The efficacy of Wnt inhibition by dnTCF-4 was confirmed by the down-regulation of β-gal (Supplementary Fig. 5b). Similar to Wnt2 shRNA, dnTCF-4 significantly reduced the expression of Nestin and the number of BrdU/DCX-double positive cells in ischemic cortex (Supplementary Fig. 5b, c).

**Fig. 4:**
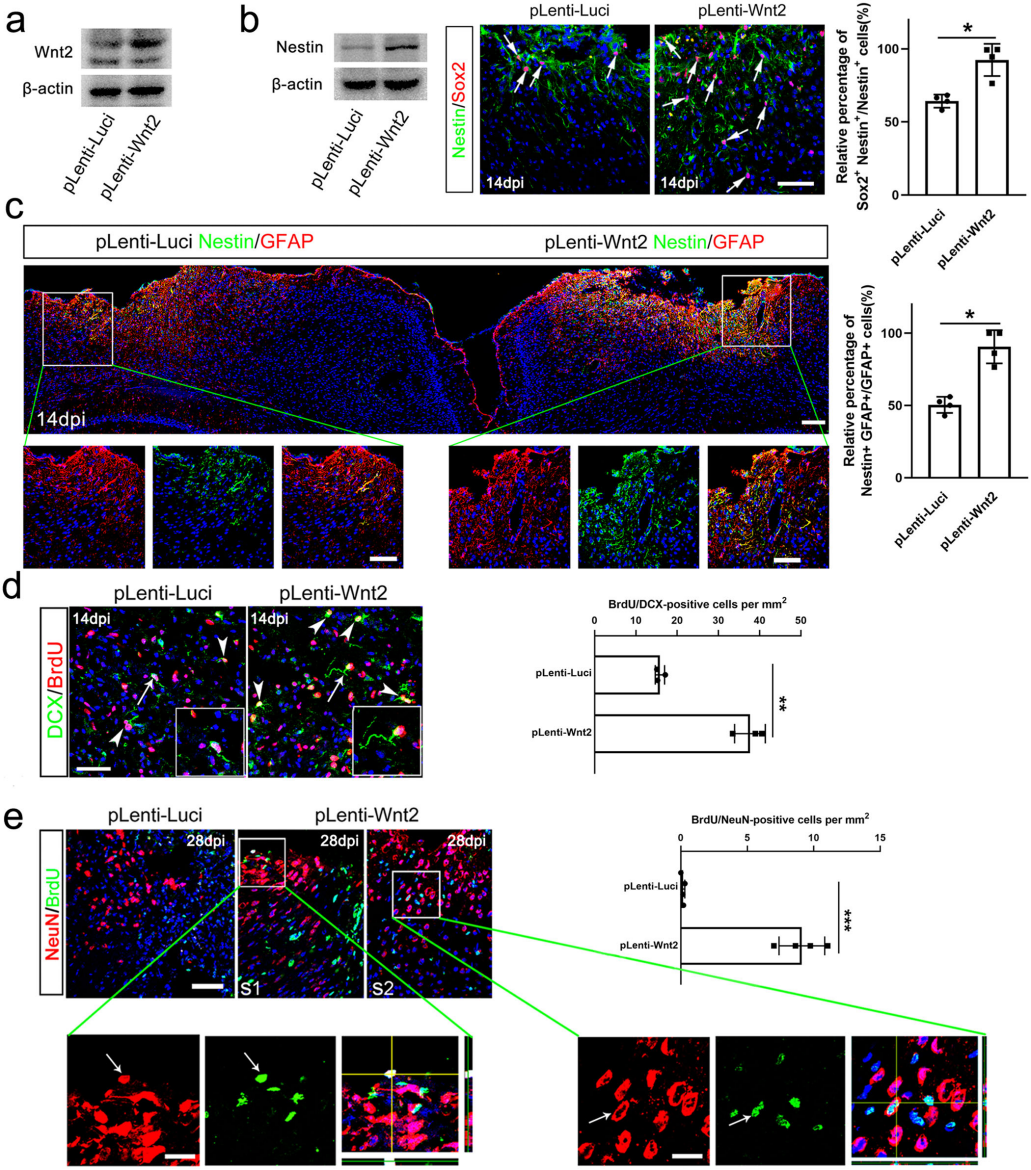
Effects of Wnt2 over-expression on astrocyte dedifferentiation and cortical neurogenesis. **a** Western-blotting of Wnt2 in ischemic cortex infected by pLenti-Luci (control) and Wnt2. **b** Western-blotting of Nestin and double-immunostaining of Nestin/Sox2 in ischemic cortex infected by pLenti-Luci (control) and Wnt2. Bar = 50 μm. *N* = 4 mice per group. **c** Representative images of Nestin/GFAP double-immunostaining in the bilateral cortices at 14 dpi with two ischemic injures infected by pLenti-Luci (left) and Wnt2 (right) respectively. Bar = 200 μm in upper panel. Bar = 50 μm in enlarged view. *N* = 4 mice per group. **d** Double-immunostaining and quantification of DCX/BrdU in ischemic cortex infected by pLenti-Luci and Wnt2. *N* = 3 mice per group. **P = 0.0075. Two-tailed Student’s *t* test. Bar = 50 μm. **e** Double-immunostaining and quantification of NeuN/BrdU in ischemic cortices infected by lentivirus expressing luciferase (control) or Wnt2. S1 and S2 are two representative sections showing one NeuN/BrdU-positive cell in each section. Arrows point to double-stained cells. Bar = 30 μm, and 5 μm in insert. ***P < 0.001. Two-tailed Student’s *t* test. *N* = 4 mice per group. Mean ± standard error (SE). Asterisks with bars connecting two groups indicate difference between these two groups.

We next assessed the effects of Wnt2 over-expression on astrocyte dedifferentiation and neurogenesis by injecting lentivirus expressing Wnt2 (Lenti-Wnt2) into ischemic cortex. Increase of Wnt2-expression was confirmed by Western-blotting (Fig. 4a). At 14 dpi when progenitor markers have declined in control virus-treated cortex, Lenti-Wnt2 sustained the high-level expression of Nestin and Sox2 (Fig. 4b, c). Further analysis showed that there was a significant increase of BrdU/DCX-positive cells in Lenti-Wnt2-infected ischemic cortex (Fig. 4d). Notably, BrdU/NeuN-positive cells, which could hardly be detected in the ischemic cortex under normal condition^30^, were found in Wnt2-overexpressing cortex with the frequency of approximately one BrdU/NeuN-positive cell in one section on average at 4 weeks after ischemia (Fig. 4e). To explore if Wnt2 could directly induce astrocyte dedifferentiation, we stimulated primary astrocytes with purified Wnt2 protein (200 ng/ml) for 72 h. Immunocytochemistry and Western-blotting revealed the notable up-regulation of Nestin by Wnt2 (Supplementary Fig. 5d–f). These data indicated that Wnt2/β-catenin signaling may serve as a local signal for triggering the dedifferentiation of reactive astrocytes and supporting neurogenesis in ischemic cortex.

As some endothelial cells also express Nestin^31^, and Wnt signaling has been reported to be involved in angiogenesis^32^, we tested if endothelial cells contributed to the changes of Nestin under the condition of Wnt signaling manipulation. The data showed that approximately 8% of Nestin-positive cells in the ischemic region were CD31-positive. Neither Wnt2 shRNA nor Wnt2 lentivirus significantly affected the percentages of CD31-positive cells within the population of Nestin-positive cells (Suppl. Fig. 5g). In addition, no significant changes of the percentages of Ki67/CD31-positive cells among CD31-positive cells were detected (Supplementary Fig. 5h). These data indicated that endothelial cells do not contribute to the dedifferentiation process observed above.

### Caspase-3 mutation abolishes both Wnt activation and astrocytes dedifferentiation in vivo

**Fig. 5:**
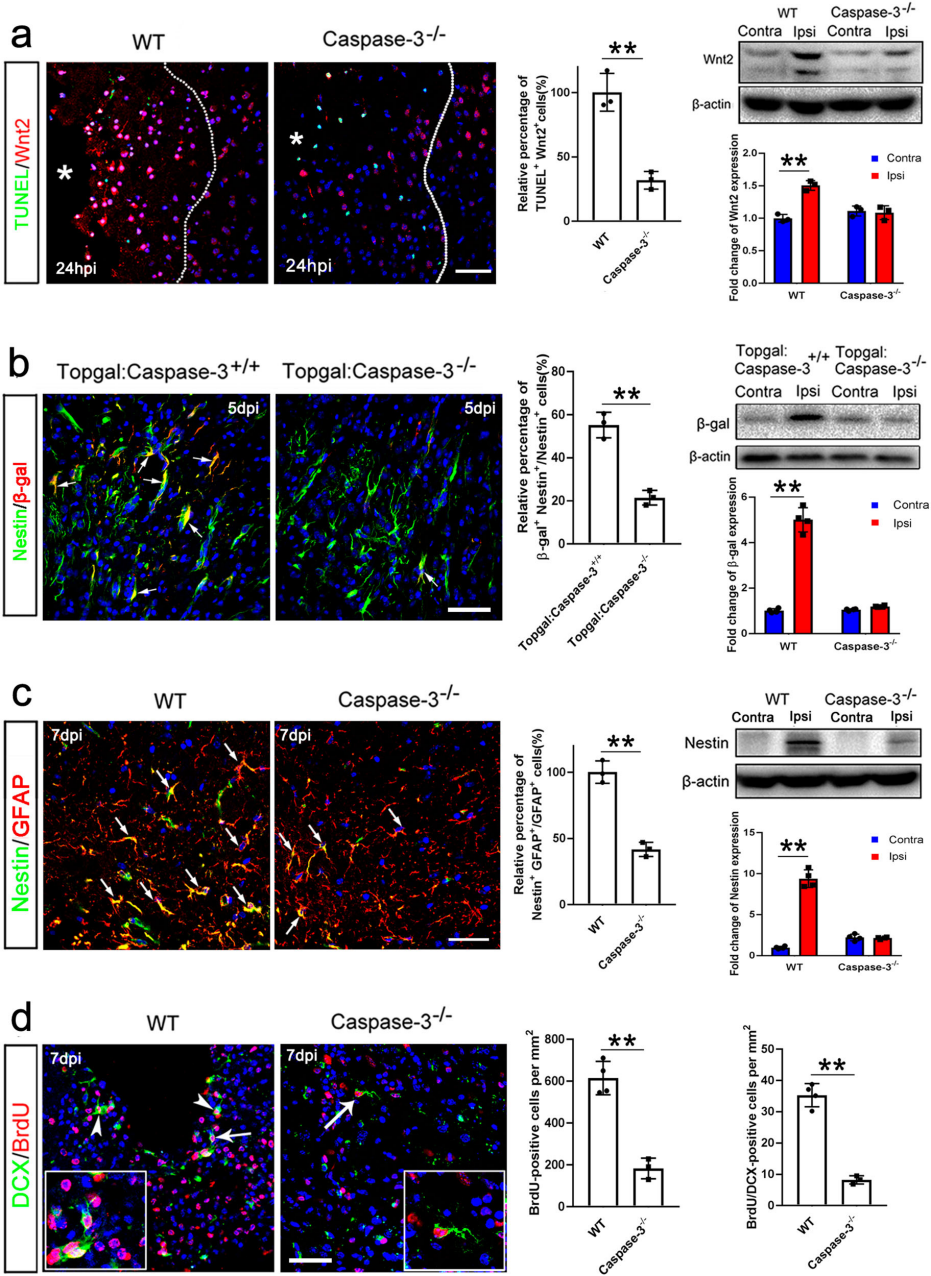
Requirement of caspase-3 for Wnt2 activation and neurogenesis in ischemic cortex. **a** Combination of TUNEL staining and Wnt2 immunostaining (left panels), and Western-blotting of Wnt2 in the ischemic cortex of WT and caspase-3^−/−^ mice (right panels). Expression of Wnt2 in the injured WT cortex is significantly increased, but remains unchanged in caspase-3^−/−^ cortex. Dashed lines indicate lesion border. Bar = 30 μm. **P = 0.0061 (left panel), **P = 0.0019 (right panel). One-way ANOVA followed by Bonferroni’s post hoc comparisons tests. *N* = 3 mice per group. **b** Representative images of Nestin/β-gal double immunostaining (left panels), and Western-blotting of β-gal in the ischemic cortex of Topgal mice with or without caspase-3 mutation at 5 dpi (right panels). The expression of β-gal in the ischemic cortex of Topgal:caspase-3^+/+^ mice is greatly enhanced, whereas it shows no notable change in Topgal:caspase-3^−/−^ mice. Bar = 30 μm. **P = 0.0096 (left panel), **P = 0.0015 (right panel). One-way ANOVA followed by Bonferroni’s post hoc comparisons tests. *N* = 3–4 mice per group. **c** Double staining of BrdU with Nestin, and Western-blotting of Nestin in the ischemic WT and caspase-3^−/−^ cortex. Notice the lower level of Nestin in the ischemic caspase-3^−/−^ cortex. **P = 0.001 (left panel), **P = 0.0012 (right panel). One-way ANOVA followed by Bonferroni’s post hoc comparisons tests. *N* = 3–4 mice per group. **d** Double staining and quantification of BrdU/DCX. The numbers of BrdU/DCX-positive cells is significantly decreased in the caspase-3^−/−^ cortex. Bars = 30 μm. **P = 0.0072 (left panel), **P = 0.0021 (right panel). Two-tailed Student’s *t* test. *N* = 3–4 per group. Arrows in (**b**, **d**) point to double-positive cells and arrowheads in (**d**) point to magnified double-positive cells. Mean ± standard error (SE). Asterisks with bars connecting two groups indicate difference between these two groups.

Since apoptotic neurons up-regulate Wnt2, we further asked whether caspase-3 ablation could affect the ischemia-induced Wnt signaling activation. Wnt2 expression in intact *caspase-3*^−*/*−^ cortex was at a similar level to that in the intact cortex of C57 WT mice (Fig. 5a), indicating that caspase-3 mutation had no significant effects on the basal expression of Wnt2. Interestingly, caspase-3 ablation significantly abolished the up-regulation of Wnt2 by ischemia (Fig. 5a). Consistently, the up-regulation of Axin2 (a Wnt target gene which faithfully reflects Wnt activation) by the ischemia was also compromised in the injured *caspase-3*^−*/*−^ cortex (Supplementary Fig. 6a). Furthermore, the expression of Wnt signaling reporter β-gal in the ischemic cortex of Topgal:*caspase-3*^−*/*−^ mice was significantly attenuated as compared to that in the lesion cortex of Topgal:*caspase-3*^*+/+*^ mice (Fig. 5b). These results indicate that caspase-3 is required for the up-regulation of Wnt2 and activation of Wnt signaling in the ischemic cortex.

We next examined the effects of caspase-3 deficiency on astrocyte dedifferentiation. As expected, there were significantly less TUNEL-positive cells in the ischemic cortex of *caspase-3*^−*/*−^ mice (Supplementary Fig. 6b). Both the expression of Nestin and the number of BrdU-positive cells was significantly reduced in the ischemic cortex of *caspase-3*^−*/*−^ mice, as compared to that of WT mice at 7 dpi (Fig. 5c, d). Western-blotting confirmed the compromised induction of Nestin in injured *caspase-3*^−*/*−^ cortex (Fig. 5c). Furthermore, BrdU/DCX-positive cells in caspase-3-deficient cortices were also decreased dramatically (Fig. 5d). On the other hand, there was no difference of Ki67/Nestin-double positive cells in the SVZ between intact WT and *caspase-3*^−*/*−^ mice at 7 dpi (Supplementary Fig. 6c), suggesting that the effects of Caspase-3 mutation on cell proliferation and Nestin expression in cortex is lesion dependent. These data indicated that caspase-3 might be involved in the ischemia-induced astrocyte dedifferentiation through activating Wnt signaling.

### Over-expressing β-catenin promotes dedifferentiation of reactive astrocytes and neurogenesis in Caspase-3^−/−^ cortex

**Fig. 6:**
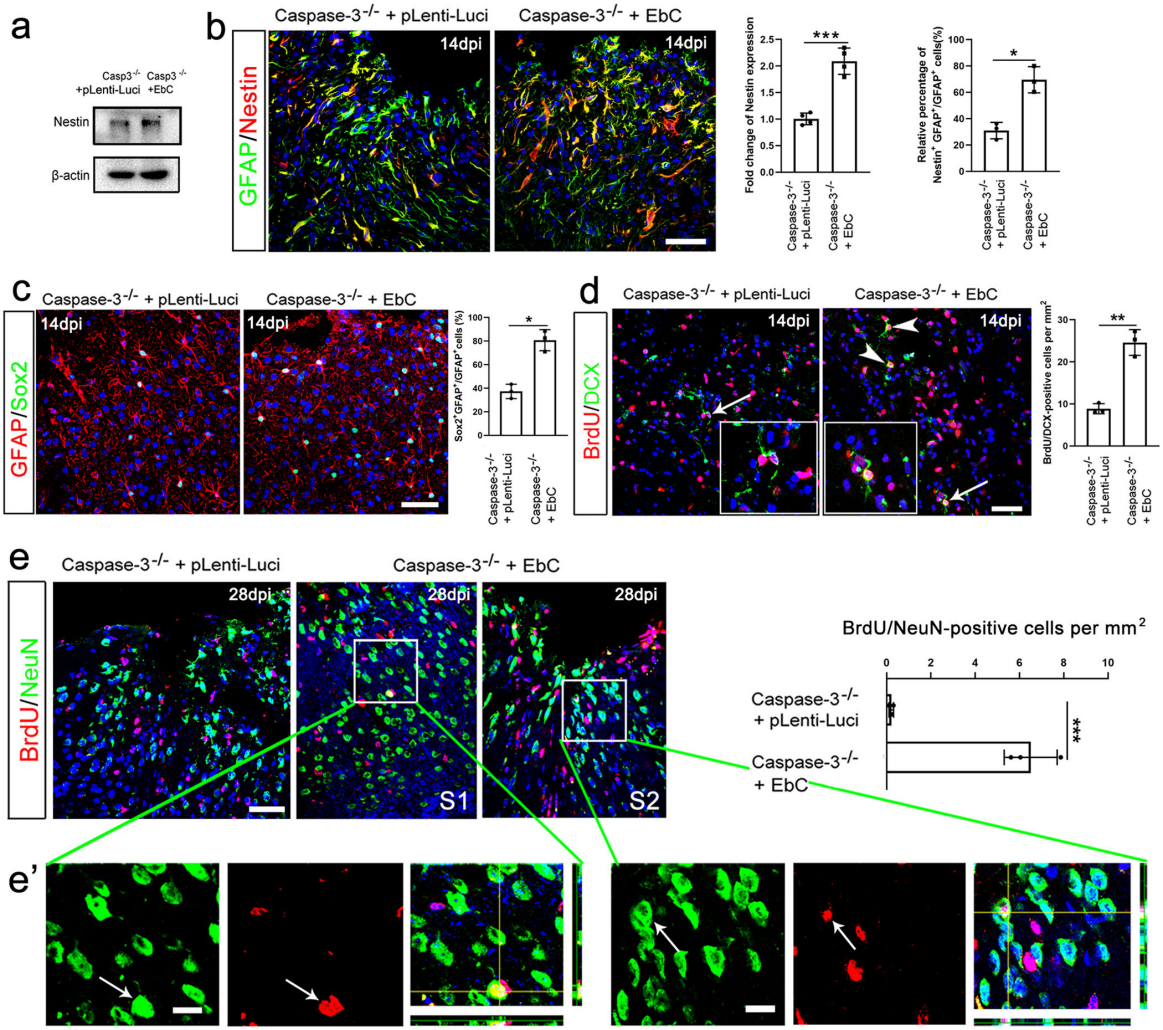
Restoration of astrocyte dedifferentiation and promotion of neurogenesis in caspase-3-deficient cortex by stabilized β-catenin. **a** Western-blotting of Nestin in ischemic caspase-3^−/−^ cortex infected by lpLenti-Luci (control) or EbC. **b** Immunostaining of GFAP/Nestin in ischemic caspase-3^−/−^ cortex infected by pLenti-Luci (control) or EbC. ***P = 0.0009 (left panel), *P = 0.0193 (right panel). Two-tailed Student’s *t* test. *N* = 3–4 mice per group. **c** Immunostaining of GFAP/Sox2 in ischemic caspase-3^−/−^ cortex infected by pLenti-Luci (control) or EbC. *P = 0.0372. Two-tailed Student’s *t* test. *N* = 3 mice per group. **d** Representative images and quantification of BrdU/DCX double immunostaining in ischemic caspase-3^−/−^ cortex infected by pLenti-Luci (control) or EbC. EbC significantly increases the number of GFAP/Sox2- and BrdU/DCX-double positive cells. Arrowheads point to double-positive cells and arrows point to magnified cells. Bar = 30 μm. **P = 0.0063. Two-tailed Student’s *t* test. *N* = 3 mice per group. **e** Double immunostaining and quantification of BrdU/NeuN -positive cells in ischemic caspase-3-deficient cortex infected with control virus or EbC. There is approximately one BrdU/NeuN-positive cell on average per section of EbC treated cortex. S1, section 1. S2, section 2. Bars = 30 μm in (**e**), 5 μm in (**e**). Arrows point to BrdU/NeuN-positive cells. ***P < 0.001. Two-tailed Student’s *t* test. *N* = 3 per group. Mean ± standard error (SE). Asterisks with bars connecting two groups indicate difference between these two groups.

We next examined whether enhancing Wnt signaling could promote astrocytic response and neurogenesis in *caspase-3*^−*/*−^ cortex by injecting lentivirus expressing stabilized β-catenin (EbC)^29^. EbC infection resulted in higher levels of Nestin and Sox2 by astrocytes in *caspase-3*^−*/*−^ cortex, in comparison with control virus (Fig. 6a–c). The number of BrdU/DCX-double positive cells was also increased in the EbC infected *caspase-3*^−*/*−^ cortex (Fig. 6d). No significant reduction of TUNEL-, TUNEL/DCX-, and TUNEL/NeuN-positive cells was observed in EbC-treated group (Supplementary Fig. 7), precluding the neuroprotective effects of EbC. Similar as Wnt2 overexpression, BrdU/NeuN-double positive cells, which could not be detected in control ischemic cortex, were found in the lesion area of EbC-infected *caspase-3*^−*/*−^ cortex (Fig. 6e).

**Fig. 7:**
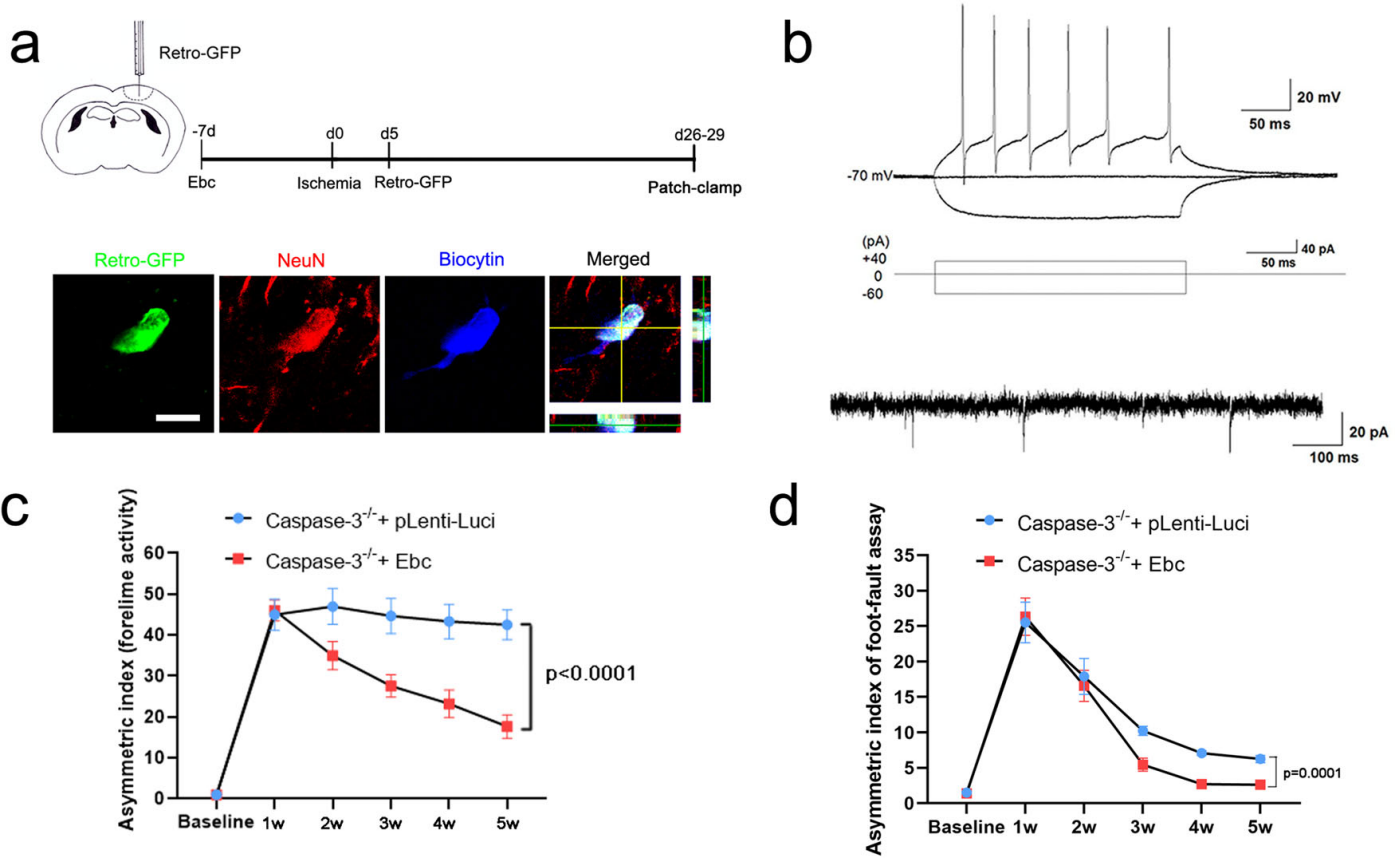
Promotion of functional recovery of caspase-3-deficient mice by stabilized β-catenin. **a** Experimental design and triple-staining of Retro-GFP/NeuN/Biocytin in patch-clamped cells. Bar = 10 μm. **b** Typical action potentials of recorded cells and typical EPSCs of the spike-firing cells. Nine (out of the 23) patched Retro-GFP-positive cells fire action potentials. Notice the spontaneous synaptic activities. **c** Glass sliding test. Stabilized β-catenin in *Caspase-3*^−*/*−^ mice significantly reduces sliding incidence of the contralateral forelimb in the glass cylinder from 3w post-injury. *N* = 6 mice per group. *P* < 0.0001 (2w, 3w, 4w and 5w). Two-way RM ANOVA. **d** Foot-fault test. Stabilized β-catenin reduces foot-fault incidence of the contralateral forelimb on a horizontally placed ladder from 3w post-injury. *N* = 6 mice per group. *P* = 0.0001 (3w, 4w, and 5w). Two-way RM ANOVA. *P* values indicate comparison of experimental group with control group at same time point. Mean ± standard error (SE).

We then examined whether the new-born neurons could have neuronal properties. Retro-virus expressing GFP was injected into EbC-treated cortex at 5–7 dpi when cell proliferation was active. Whole cell patch-clamp recording of Retro-GFP positive cells was performed at 26–29 dpi (Fig. 7a). Biocytin was injected to make sure the cells recorded were Retro-GFP labeled neurons (Fig. 7a). Among the 23 successfully patched cells, action potentials were recorded from 9 cells which had an average input resistance of 415.4 ± 87.3 MΩ and resting membrane potential of −56.8 ± 2.5 mV (Fig. 7b). Three Retro-GFP-labeled cells showed spontaneous excitatory post-synaptic currents (sEPSCs) (Fig. 7b). To test whether EbC could enhance functional recovery in *caspase-3*^−*/*−^ mice, we performed focal ischemia in the forelimb sensorimotor cortex and evaluated the locomotion recovery by foot-fault test and spontaneous forelimb activity. The results showed that stabilized β-catenin significantly improved the spontaneous activity of contralateral (to the injured cortex) forelimb and reduced the foot-miss of forelimbs on a horizontally placed ladder from 3 weeks after injury (Fig. 7c, d). These results indicated that activating Wnt/β-catenin signaling not only restores astrocyte dedifferentiation but also promotes neurogenesis and functional recovery in the ischemic caspase-3-deficient mice.

### Apoptotic neurons up-regulate Wnt2 protein via IRES-mediated alternative translation

**Fig. 8:**
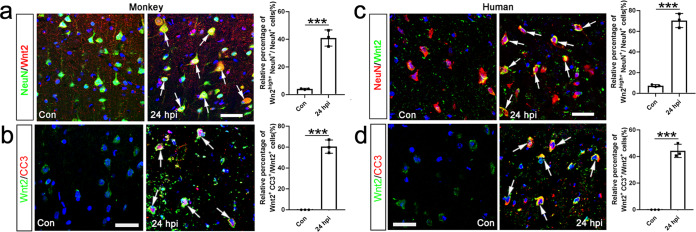
Expression of Wnt2 in ischemic cortex of Macaca Mulatta and human cortex. **a**, **b** Double-immunostaining of NeuN/Wnt2, Wnt2/CC3 in control and injured cortex of Macaca Mulatta at 24 hpi. Bar = 30 μm. **c**, **d** Double-immunostaining of NeuN/Wnt2, Wnt2/CC3 in human cortical autopsy tissue (control and 24 hpi). Bar = 30 μm. Notice the higher Wnt2-immunoreactivity in CC3-positive cells in both Macaca Mulatta and human cortex. Arrows point to representative double-positive cells. ***P < 0.001. Two-tailed Student’s *t* test. *N* = 3 samples from 2 monkeys or patients per group. Mean ± standard error (SE). Asterisks with bars connecting two groups indicate difference between these two groups.

Above data demonstrated that apoptosis-activated Wnt2 triggered astrocyte dedifferentiation in mouse. We then asked whether this apoptosis-induced Wnt2 expression could occur in other species. We analyzed the cortical sample of Macaca Mulatta and human patients at 24 h after ischemia. Relative weak Wnt2-immunoreactivity was detected in neurons under normal condition while strong Wnt2-immunoreactivity (Wnt2^high^) was observed in the ischemic cortex of both Macaca Mulatta and human (Fig. 8a, c). Double-immunostaining of NeuN with cleaved caspase-3 (CC3) revealed that many neurons underwent apoptosis in the ischemic cortex (Supplementary Fig. 8). Similar as that in mice, most of the Wnt2^high^ cells in the ischemic cortex of Macaca Mulatta and human expressed CC3 (Fig. 8b, d). These data indicated that this apoptosis-induced Wnt2 up-regulation is a conserved phenomenon.

**Fig. 9:**
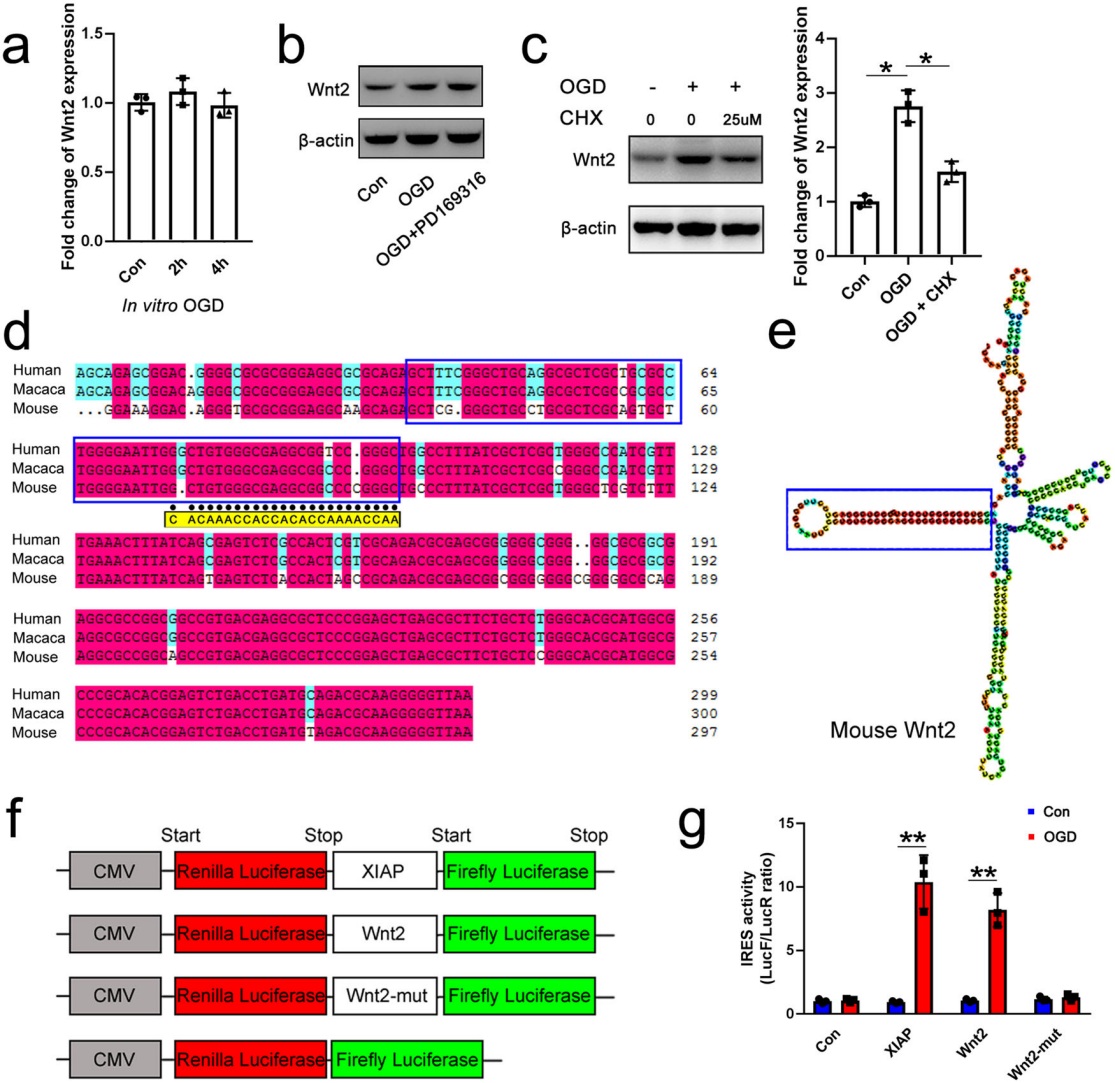
IRES-mediated Wnt2 protein translation. **a** Real time RT-PCR of Wnt2 in OGD treated neurons. **b** Western-blotting of Wnt2 in apoptotic neurons treated with PD169316. *N* = 3 batches of cells. **c** Western-blotting and quantification of Wnt2 in apoptotic neurons treated with CHX. Notice that OGD does not induce Wnt2 mRNA transcription and that CHX blocks OGD-induced Wnt2 protein up-regulation. *P_con vs OGD_ = 0.011, *P_OGD VS OGD+CHX_ = 0.031. One-way ANOVA followed by Bonferroni’s post hoc comparisons tests. *N* = 3 batches of cells. **d** Sequence alignment of mouse, Macaca Mulatta and human Wnt2-5UTR. **e** Secondary structure of mouse Wnt2-5UTR. Blue frames in (**d**) and (**e**) show the largest stem-loop and the corresponding sequence. The sequence underlined by black dots (in **d**) was replaced by the sequence below highlighted with yellow color to make Wnt2-5UTR mutant construct in (**f**). **f**, **g** Bicistronic luciferase reporter assay of XIAP-5UTR, Wnt2-5UTR, and Wnt2-5UTR mutant under control and OGD condition. **P_XIAP_ = 0.0022, **P_Wnt2_ = 0.0019. One-way ANOVA followed by Bonferroni’s post hoc comparisons tests. *N* = 3 batches of cells. Notice that Wnt2-5UTR initiates OGD-induced Wnt2 translation and mutation at the largest loop abolishes this translation. Mean ± standard error (SE). Asterisks with bars connecting two groups indicate difference between these two groups.

We next probed how apoptotic neurons up-regulated Wnt2. qPCR revealed no changes of Wnt2 mRNA under apoptotic conditions both in vitro and in vivo (Fig. 9a, Supplementary Fig. 9a), suggesting that post-transcription mechanism may account for the up-regulation of Wnt2 protein. Intriguingly, 26 S proteasome activator PD169316 did not reduce the levels of Wnt2 protein (Fig. 9b), while protein translation inhibitor CHX significantly attenuated the OGD-induced Wnt2 up-regulation (Fig. 9c), indicating that apoptotic neurons may up-regulate Wnt2 at the level of protein translation.

In apoptotic cells, the translation of most proteins is suppressed except for few proteins which are crucial for cell survival or apoptosis, such as insulin-like growth factor (IGF) and X-linked inhibitor of apoptosis (XIAP)^33,34^. The “escaped” protein translation is achieved mainly by cap-independent translation, and in most cases, through the internal ribosome entry site (IRES) sequence in the 5ʹ-untranslated region (5UTR) of the mRNA^35^. Sequence analysis revealed that the Wnt2-5UTR of mouse, Macaca Mulatta and human shared highly conserved sequence (Fig. 9d) which could form IRES-like secondary structure (Fig. 9e). To test the function of Wnt2-5UTR, we inserted the Wnt2-5UTR into a bicistronic reporter vector and examined the activity of Wnt2-5UTR in MCF-7 cells which normally express Wnt2 and up-regulate Wnt2 protein upon OGD treatment (Fig. 9f, Suppl. Fig. 9b). OGD treatment induced dramatic expression of luciferase downstream of Wnt2-5UTR as positive control XIAP-5UTR did (Fig. 9g). Mutation of conserved sequence in the largest stem-loop of Wnt2-5UTR significantly abolished this apoptosis induced reporter expression (Fig. 9d, e, and g). These data indicated that apoptotic neurons may up-regulate Wnt2 protein through IRES-mediated protein translation.

### Death-associated protein 5 mediates Wnt2 up-regulation and astrocyte dedifferentiation

**Fig. 10:**
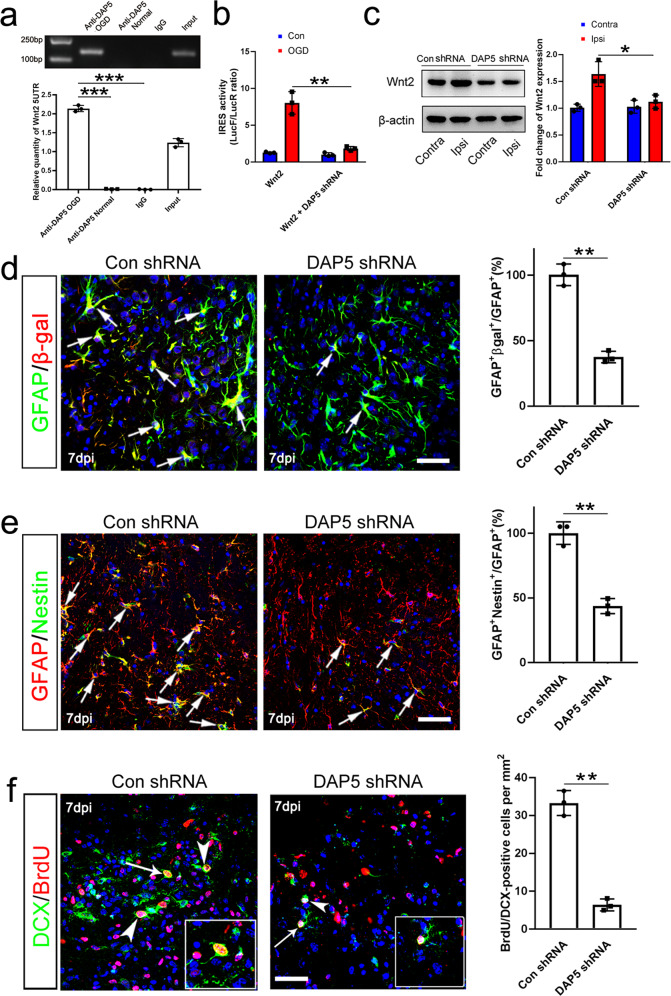
Involvement of DAP5 in Wnt activation and astrocyte dedifferentiation. **a** RIP assay. Notice that DAP5 could bind to the Wnt2 5UTR under OGD. ***P < 0.001. One-way ANOVA. *N* = 3 batches of cells. **b** IRES activity of Wnt2 5UTR with or without DAP5 shRNA under OGD. DAP5 shRNA effectively blocked the OGD-induced IRES activity. **P = 0.0052. One-way ANOVA followed by Bonferroni’s post hoc comparisons tests. *N* = 3 batches of cells. **c** Western-blotting of Wnt2 in the contralateral and ipsilateral cortex infected by lentivirus expressing DAP5 shRNA or control shRNA. *P = 0.039. One-way ANOVA followed by Bonferroni’s post hoc comparisons tests. *N* = 3 mice per group. **d** Double-immunostaining and quantification of GFAP/β-gal in ischemic cortex treated by control shRNA and DAP5 shRNA. Notice the attenuated expression of β-gal in DAP5 shRNA treated cortex. **P = 0.0081. Two-tailed Student’s *t* test. *N* = 3 mice per group. **e** Double-immunostaining and quantification of GFAP/Nestin in ischemic cortex treated by control shRNA and DAP5 shRNA. Ischemia-induced Nestin was reduced in DAP5 shRNA treated cortex. **P = 0.0097. Two-tailed Student’s *t* test. *N* = 3 mice per group. **f** Double-immunostaining of DCX/BrdU in ischemic cortex treated by control shRNA and DAP5 shRNA. Notice the decreased number of DCX/BrdU-positive cells in DAP5 shRNA treated cortex. Arrowheads point to double-positive cells. Arrows points to magnified cells. Bar = 30 μm. **P = 0.0063. Two-tailed Student’s *t* test. *N* = 3 mice per group. Mean ± standard error (SE). Asterisks with bars connecting two groups indicate difference between these two groups.

In apoptotic cells, death associate protein 5 (DAP5) is one of the key protein which recruits ribosome to the IRES sequence of target mRNA and facilitates translation^36,37^. We then tested whether this mechanism was involved in the apoptosis-induced Wnt2 up-regulation. Western-blotting showed that DAP5 was up-regulated in ischemic cortex (Supplementary Fig. 9c). RNA immunoprecipitation showed that DAP5 bound to the 5UTR of Wnt2 under apoptotic condition (Fig. 10a). Pretreating primary neurons with lentivirus expressing DAP5 shRNA significantly attenuated the translation initiating activity of Wnt2-5UTR under OGD (Fig. 10b, Supplementary Fig. 9d). Local injecting lentivirus expressing DAP5 shRNA to cortex or cultured neurons significantly attenuated the ischemia/apoptosis-induced Wnt2 up-regulation both in vivo and in vitro (Fig. 10c, Supplementary Fig. 9e). Accordingly, the ischemia-induced expression of Wnt reporter β-gal in astrocytes was significantly reduced in the cortex of Topgal mice infected by lentivirus expressing DAP5 shRNA (Fig. 10d).

We next asked whether DAP5 knockdown affected the dedifferentiation of astrocytes and neurogenesis post cerebral ischemia. Double-immunostaining showed that the expression of Nestin and Sox2 was significantly reduced in DAP5 shRNA pre-treated cortex while the expression of GFAP remain not changed (Fig. 10e, Supplementary Fig. 9f). The number of BrdU/DCX-positive cells was significantly reduced in DAP5 shRNA pre-treated cortex (Fig. 10f). These data suggested that DAP5 shRNA can partially phenocopy the effects of Wnt2 knockdown in terms of astrocytes dedifferentiation and cortical neurogenesis.

## Discussion

**Fig. 11:**
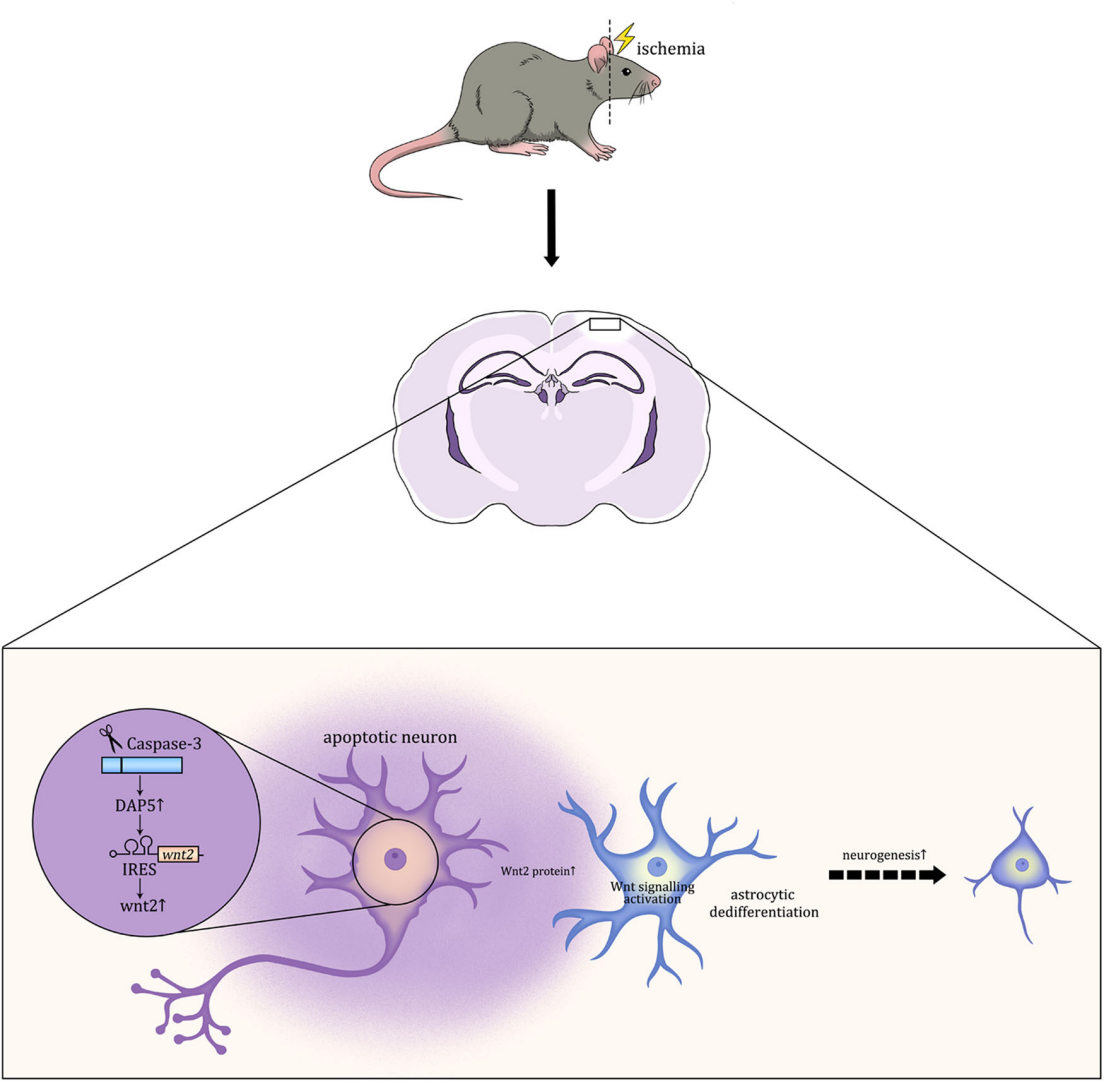
Schematic graph of the major finding. Following cerebral ischemia, apoptotic neurons up-regulate Wnt2 protein via IRES mediated protein translation. Released Wnt2 activates Wnt/β-catenin signaling in reactive astrocyte and triggers astrocytic dedifferentiation, which facilitated cortical neurogenesis.

In the present study, by using a Wnt signaling reporting mouse line Topgal, we first demonstrated the activation of canonical Wnt signaling in reactive astrocytes following focal ischemia. By lentivirus-mediated Wnt2 silencing and Wnt2 over-expression, we demonstrated a key role of Wnt2 in the dedifferentiation response of astrocytes and post-ischemic neurogenesis. Interestingly, the data from caspase-3 deficient mice revealed the requirement of caspase-3 for this ischemia-induced Wnt2 up-regulation and astrocyte dedifferentiation. In the end, we demonstrated that the up-regulation of Wnt2 was mediated by IRES-mediated protein translation (Fig. 11).

The present study focused on the in vivo dedifferentiation response of reactive astrocytes and neurogenesis in cortex after ischemia. The up-regulation of neural progenitor markers by reactive astrocytes has been reported^6^. Previous studies have demonstrated that SVZ progenitors contributed to the neurosphere-forming reactive astrocytes in cerebral cortex^22,38^. However, how the local reactive astrocytes up-regulate progenitor markers remained to be explored. Our observation that local delivery of Wnt2 shRNA in ischemic cortex effectively reduced Nestin expression in reactive astrocytes suggested a key role of Wnt2 in the response of local astrocytes, because the migrating SVZ progenitors had already expressed Nestin. The induction of Ngn2 by ischemia and the abolishment of this induction by Wnt2 shRNA supported the importance of Wnt2 in astrocyte dedifferentiation. Previously, researchers have speculated that reactive astrocytes might be potentially neurogenic. However, the in vivo evidence for neurogenesis from cortical astrocytes is still not sufficient and recent study challenged previous finding that NeuroD1 and PTB1 could induce in vivo reprogramming of astrocytes into neurons^18,39^. The increase of DCX-positive neuroblasts and BrdU/NeuN-positive mature neurons by Wnt2 and β-catenin overexpression was important and interesting. This added hints to the idea that the local dedifferentiation of Wnt-activated astrocytes may be neurogenic, or alternatively, provide a supporting microenvironment for the SVZ-derived neurogenesis. Therefore, whether dedifferentiating astrocytes could generate neurons needs to be investigated by more well-designed fate-mapping experiments. Nevertheless, the worsen of forelimb activity by Wnt2 knockdown suggested that astrocytic dedifferentiation was beneficial for functional recovery following ischemia. The promotion of functional recovery by over-expressing β-catenin suggested that amplifying Wnt signaling could be utilized for treating cerebral ischemia in the future.

It is very interesting that neuronal Wnt2 up-regulation and astrocytic dedifferentiation is caspase-3 dependent. Injury induced-Wnt activation has been documented in multiple tissues^40^, while how injury activates Wnt signaling remains unclear. Apoptosis, probably one of the most conserved ultimate cell fate upon injury, was thought to account for the injury-induced Wnt activation in low animals, for example, in hydra^41^. In mammalian, two studies in skin and tumor tissues, reported that apoptotic cells induce wound healing^42^ and tumor cell repopulation^43^ by releasing PGE_2_, a potential Wnt signaling activator^44^. In cerebral cortex, it is still a controversial topic concerning whether apoptosis could induce cortical neurogenesis^45,46^. Recently, a study reported that the proliferation of microglia is apoptosis-coupled^47^. Our data revealed that apoptotic neurons released Wnt2 as an endogenous reparative signal. This apoptosis-induced Wnt activation may be extended to other systems. As conventional *Casapse-3* knockout mice and lenti-virus expressing Wnt2 shRNA/Wnt2 were used in this study, the involvement of caspase-3 and Wnt2 in other cell types is not excluded.

The IRES-mediated Wnt2 translation is intriguing. It is well accepted that cap-dependent translation is inhibited during apoptosis and only a few proteins essential for cell survival or apoptosis can be translated through a cap-independent mechanism^33^. DAP5 plays a key role in the protein translation of apoptotic cells, which requires the IRES structure in the 5UTR of target protein to recruit ribosome^36,48^. Our data demonstrated that the apoptotic neurons up-regulated Wnt2 via DAP5-dependent translation, and the mutation of the IRES sequence in the 5UTR of Wnt2 abolished the translation of Wnt2. The high homology of Wnt2 5UTR among mouse, Macaca Mulatta and human suggested that this apoptosis-induced Wnt2 up-regulation may be a conserved mechanism for ischemia-induced astrocyte dedifferentiation. The attenuation of astrocyte dedifferentiation by DAP5 shRNA substantiated the role of DAP5 in this process. Targeting Wnt2 5UTR or DAP5 may shed insight on developing new treatment for stroke.

## Methods

### Mice and reagents

The Topgal mice (Jax stock No. 004623), caspase-3^−/−^ mice (Jax stock No. 006233) and ROSA-DTA mice (Jax stock No. 009669) were obtained from Jackson lab. Topgal:caspase-3^−/−^ mice were breed by crossing Topgal mice with caspase-3^−/−^ mice for over two generations. The generation of Nestin-CreER mice was described previously^49^. Nestin-CreER:ROSA-DTA mice were breed by crossing Nestin-CreER mice with ROSA-DTA mice. All mice experiments were carried out under protocols approved by the Animal Care and Use Committees of Fourth Military Medical University.

Lentiviral vectors expressing stabilized β-catenin (EbC) and dominant-negative form of TCF-4 (dnTCF-4, also called EdTC) were kindly gifted by Dr. Fuerer Christophe and Dr. Roel Nusse (Stanford University). Retrovirus expressing GFP, lentivirus expressing Wnt2 shRNA, Wnt2, and DAP5 shRNA were purchased from Obio Biotech, Shanghai. Pan-caspase inhibitor v-ZAD (Cat: FMK001) was purchased from R & D System. Tamoxifen (Cat: T5648) was purchased from Sigma.

### Mice focal ischemia model

For all in vivo experiments, mice were anesthetized by using vaporizer for isoflurane (R583S, RWD). Focal photochemic ischemia was conducted as the following. Rose bengal (Cat: 330000, Sigma) was injected via tail vein at 25 mg/kg. For most experiments, a skull window ranging from 0.8 to 2 mm posterior to the Bregma, and 2 mm right to the midline was carefully made. For the behavior tests, the skull widow was made from 0.3–2.3 mm anterior to the Bregma, and 0.5–3.0 mm right to the midline. Brains were illuminated for 10–12 minutes using a cold light source (Zeiss FL1500 LCD). For neuroblasts detection, BrdU (100 mg/kg; Cat: B5002, Sigma) injection was started 12 h after ischemia, once a day for 6 days. Mice were sacrificed at 7 or 14 dpi.

### Mice treatment

Lentivirus expressing control shRNA, Wnt2 shRNA, DAP5 shRNA or dnTCF-4 ((1–3)×10^6^ in 500 nl) was injected into the cortex 9 days before ischemia. Wnt2 shRNA sequences and DAP5 shRNA sequences are provided in the supplementary information.

For Wnt2 and β-catenin over-expression, lentivirus expressing Wnt2, EbC or control virus expressing luciferase ((1–3) × 10^6^ in 500 nl) was injected into the cortex 9 days before ischemia. For DCX/BrdU-staining, BrdU was injected once per day from 3 to 10 dpi, and mice were sacrificed at 14 dpi. For NeuN/BrdU-staining, BrdU was injected once per day from 3 to 17 dpi, and mice were sacrificed at 28 dpi.

For neural progenitor depletion, Tamoxifen (TAM, 80 mg/kg) was intraperitoneally injected for 3 consecutive days. After 2 days interval, ischemia was made and mice were sacrificed at 7 dpi.

### Monkey focal ischemia model

Before ischemia, a healthy male Macaca Mulatta of 19 years old was anaesthetized by ketamine for anesthesia induction and pentobarbital sodium for anesthesia maintenance. Then, T1 and T2 MRI scanning were performed on both left and right motor cortex. According to the localization of MRI scanning, endothelin (Cat: E7764, Sigma, 5 ug/ul) was injected into 3 points of one side motor cortex with intervals of 5 mm (5 μl per point, 4 mm in depth) to induce ischemia. At 24 h post ischemia, the monkey was perfused through heart by 4% paraformaldehyde phosphate buffer (PFA, Cat: P0099, Beyotime). The brain tissue was dissected immediately after perfusion and post-fixed by 4% PFA for about 2 h. All the procedure and protocol of monkey experiments were performed under the approval of the Animal Care and Use Committees of Kunming Institute of Zoology, Chinese Academy of Sciences (animal ethic review number: IACUC19009).

### Human brain samples

The study protocol involving human brain samples was reviewed and approved by the Ethics Committee of Xijing hospital (KY20202003). Ischemic cortical samples were obtained from the post-stroke areas in two patients who met the following criteria: (1) requiring both decompressive craniectomy and partial lobectomy for diffuse cerebral infarction at 18–26 h post-ischemia; (2) >20 years of age; and (3) willing to provide written informed consent. Control cortical biopsy samples were obtained from a patient with severe traumatic brain injury. This patient underwent decompressive craniectomy and the samples was resected from the peri-contusional area of the temporal cortex. The diagnosis of cerebral infarction and trauma was made by neurosurgeons in the department of neurosurgery of Xijing hospital according to physical examination and neuroimaging (CT and MRI). Excluding criteria were set as: (1) presence of malignant tumor, (2) presence and history of major infectious diseases, and (3) patients judged as unsuitable by the attending doctor.

### Western-blotting

For the Western-blotting of cortical tissue, cortical samples including lesion center and 3–5 mm surrounding tissues were dissected out. Each sample was homogenized in RIPA buffer (Cat: P0013B, Beyotime) for about 20 min and incubated for another 40 min on ice, then centrifuged at 12,000 × *g* at 4 °C. For the Western-blotting of primary cells, cells were rinsed by PBS, collected, and then lysed with RIPA buffer. Protein sample was boiled before sodium dodecyl sulfate polyacrylamide gel electrophoresis. The proteins were electrotransferred to polyvinylidene difluoride membrane and reacted with primary antibodies (as described in Supplementary materials) overnight at 4 °C, then incubated with corresponding secondary anti-mouse, anti-rabbit, anti-goat, or anti-rat IgG peroxidase (1:5000) at room temperature for 50 min. The bands were visualized by an ECL kit (Cat. WBULS0100, Millipore). For tissues and cells, β-actin and LMNB1 were used as cytoplasmic and nuclear internal control respectively. For the conditioned medium, BSA was used as internal control. All blots and gels derived from same experiment and were processed in parallel.

### Real-time RT-PCR

For real-time RT-PCR, total RNA was extracted using Trizol agent (Cat: 15596026, Invitrogen). The RNA samples were treated with DNase I (Cat: 18047019, Invitrogen) to exclude the contamination of genomic DNA. After reverse transcription, semi-quantitative PCR was carried out. The levels of target mRNA were normalized to the mRNA levels of the housekeeping gene *Gapdh* to allow comparison using the 2^−ΔΔCt^ method. The primer information was listed in supplementary materials.

### RNA immunoprecipitation (RIP) assay

When cells reach 80% confluence, MCF7 cells were treated with OGD for 12 h and cultured for another 24 h before lysis. Cells treated with or without OGD were collected and the subsequent RIP assay was performed according the manual of RIP kit (Geneseed Biotech Co., Cat. No. P0101). Anti-DAP5 (50 μg, sc-137011, Santa Cruz Biotech) was added to precipitate RNA-protein complex. Anti-mouse IgG (50 μg) was used as control. The RNA isolated from all groups (anti-DAP5-OGD, anti-DAP5-normal, IgG and input) were used to perform real time PCR. The primer sequences targeting the Wnt2-5UTR were as the following: Wnt2-5UTR-F: AAGAAGATGGGAAGCGCCAA; Wnt2-5UTR-R: ACCGCTTTACAGCCTTCCTG. Relative quantification was made by 2^−ΔΔCt^ method.

### Enzyme-linked immunosorbent assay (ELISA)

The concentrations of Wnt2 in the conditioned medium of control neurons, neurons treated with OGD, and neurons treated with OGD and Wnt2 shRNA were determined using an ELISA kit (Cusabio, CSB-EL026133MO) according to the manufacturer’s instruction. The absorbance was measured by a multimode microplate reader (TECAN, infinite M200) at 450 nm.

### Behavioral tests

Glass sliding and foot-fault tests were used to assess the asymmetry of forelimb-use^50,51^. The animals were videotaped in a transparent glass cylinder for 10 min. The contact numbers of the sliding movements of each forelimb at the wall of the cylinder for every spontaneous stand-up were scored. Percentage of sliding was calculated by the following formula: the number of sliding/(number of contact + number of sliding) ×100. The asymmetry index was derived by subtracting the percentage of ipsilateral sliding from the percentage of contralateral sliding.

For foot-fault tests, animals were allowed to walk on a horizontally placed ladder (1.5 cm width between rungs) for 10 minutes. A step was considered a foot-fault if it was not providing support or a slip/miss occurred. Foot-faults for each limb were counted and compared to the overall step number taken by that limb. The observer was blind to experimental design and mouse genotypes. Thus, percentage of foot-faults was calculated by: the number of foot-faults/(number of steps + number of foot-faults) ×100. The asymmetry difference was derived by subtracting the percentage of ipsilateral foot-faults from the percentage of contralateral foot-faults.

### Electrophysiological recording

Retrovirus expressing GFP (Retro-GFP) was injected in caspase-3^−/−^ cortex with overexpression of EbC at 5–7 dpi when cell proliferation was active and patch-clamp was performed at 21–24 dpi. Neurons in injured cortex expressing GFP were selected for recording. The electrode was filled with potassium based intrapipette solution with a resistance of about 7–9 MΩ. Cells with series resistance more than 30 MΩ at any time during the recordings were discarded. Pipette offset current was zeroed immediately prior to contacting the cell membrane. The data were sampled at 20 kHz for current-clamp recordings and 10 KHz for voltage-clamp recording with pCLAMP 9 software and digidata 1322 (Axon Instruments). Action potentials were recorded in the presence or absence of 1 μM TTX (Cat:203732, Sigma).

### Immunohistochemistry

Mice were sacrificed at each time point and perfused intracardially with 4% cold PFA. The brain tissue was dissected immediately after perfusion and post-fixed by 4% PFA for about 2 h at 4 °C. Then, the tissues were cryoprotected by 25% sucrose (Cat: S0389, Sigma). Serial coronal sections were prepared on a cryostat. Primary antibodies were incubated at room temperature overnight (detailed information was included in Supplementary information). Before adding primary antibodies, antigen retrieval was conducted for Wnt2 immunostaining, and HCl treatment (2 N HCl, 30–35 min at 37 °C) was performed for BrdU staining. Corresponding secondary antibodies conjugated with Alexa Fluor 594 (donkey anti-mouse, anti-rabbit, anti-goat or anti-guinea pig IgG, 1:800. Antibody registry ID: AB_2340432, AB_2340854, AB_2340621, AB_2340474. Jackson ImmunoResearch), Alexa Fluor 488 (donkey anti-mouse, anti-rabbit, or anti-goat IgG, 1:800. Antibody registry ID: AB_2313584, AB_2338845, AB_2340428. Jackson ImmunoResearch), Alexa Fluor 647 (donkey anti-rabbit IgG, 1:800. Antibody registry ID: AB_2492288. Jackson ImmunoResearch) were incubated with sections for 4 h at room temperature in dark.

TUNEL staining was performed according to the manual of DeadEND^TM^ TUNEL system (Cat: G3250, Promega). For combination of TUNEL staining with immunohistochemistry, TUNEL staining was performed first, followed by individual immunostaining.

### Culture of cortical neurons and astrocytes, induction and inhibition of apoptosis, and collection of conditioned medium

Cortical neurons were isolated from mouse embryos at embryonic days 15.5–16.5. For culturing caspase-3^−/−^ neurons and their WT controls, neurons were isolated from P0 pups. Astrocytes were primary isolated from P2–4 pups, and purified by shaking the flask at 100 × *g* overnight at 37 °C. Primary neurons were treated by oxygen-glucose deprivation (OGD) which meant that neurons were cultured with 95% N_2_ + 5% CO_2_ and DMEM free of glucose for 12 h to induce apoptosis. For inhibition of apoptosis, 25 μM z-VAD (Cat: FMK001, R&D System) was added into the culture 2 h before OGD and maintained throughout of OGD. Conditioned medium from normal cultured neurons (NNCM), apoptotic neurons (ANCM) and apoptosis-inhibited neurons (AICM) was collected at 18–20 h after OGD and centrifuged at 700 × *g* for 10 min. Supernatants were stored in −70 °C until use.

### In situ hybridization

Antisense digoxigenin-labeled RNA probes for Wnt2, Wnt7a, Wn9a and Wnt10a were synthesized. All the probes were made by RT-PCR based in vitro transcription. The primer information is as the following: Wnt2: TGTACTCTGAGGACATGCTGGCT, CTTATGTGCAGCAGGTGGTTC; Wnt7a: TCAGCCTGGGCATAGTCTACCTCC, TTCTCCTCCAGGATCTTCCGACCC; Wn9a: CAAGTTTGTCAAGGAGTTCCTGG, TGTTGTTTGTAACCCTGTGCC; Wnt10a: CTGTTCTTCCTACTGCTGCTGGCTGC, ATGTTCTCCATCACCGCCTGCC. 100 ng/ml of probes were used for hybridization. After hybridization and incubation with anti-digoxigenin antibody (Ref. 11093274910, Roche), color was development with alkaline phosphatase mediated reaction.

### Luciferase assay of Wnt2-5UTR IRES activity

MCF-7 cells were routinely maintained. Wnt2-5UTR was PCR amplified from genome DNA of mice liver and cloned into plasmid pR-F. Mutations within the conserved stem-loop region of Wnt2-5UTR were introduced by overlap extension PCR. The resulting mutation was confirmed by Sanger sequencing. Twenty-four hours after plasmids transfection, OGD was performed and lasted for 24 h. Luciferase assay was conducted using the luciferase reporting kit (Cat: E1910, Promega) after 8 h’s reoxygenation using GloMax. The ratio of LucF/LucR was used to indicate the activity of IRES. XIAP-5UTR was used as positive control^52^.

### Statistical analysis

For in vivo proliferation and neurogenesis, the numbers of immuno-double labeled cells were counted from every eighth section of ischemic region and at least three mice included for each comparison. Cell counting was performed by an investigator who was blind to experimental design and mouse genotypes. Data are presented as mean ± standard error. Statistical comparisons were made using the Student’ *t* test or analysis of variance (ANOVA) with Bonferroni’s post hoc comparisons tests. *P* values less than 0.05 were considered as statistically significant.

### Reporting summary

Further information on research design is available in the Nature Research Reporting Summary linked to this article.

## Supplementary information

Supplementary Information

Reporting Summary

## Data Availability

All data needed to evaluate the conclusions in the paper are present in the paper and/or the Supplementary Materials. Additional data related to this paper may be requested from the authors.
