# Supplementary material for: IRES-mediated Wnt2 translation in apoptotic neurons triggers astrocyte dedifferentiation
Source: NPJ Regen Med. 2022 Sep 2;7:42. doi: 10.1038/s41536-022-00248-1 (PMC9440034; doi:10.1038/s41536-022-00248-1)

**Title:** IRES-mediated Wnt2 translation in apoptotic neurons triggers astrocyte dedifferentiation

**Authors:** Hong Fan[1,9]†, Jialei Yang[2]†, Kun Zhang[1]†, Junling Xing[11]†, Baolin Guo[1], Honghui Mao[1], Wenting Wang[1], Yingzhou Hu[3], Wei Lin[4], Ying Huang[5], Jian Ding[6], Caiyong Yu[1], Fanfan Fu[1], Li Sun[1], Jing Wu[3], Youyi Zhao[1], Wenbin Deng[7], Chengji Zhou[8], Mengsheng Qiu[10], Shengxi Wu[1*], Yu-Qiang Ding[5*], Yazhou Wang[1*]

## Supplementary information

**(1) Wnt2 shRNA target sequences:**

Wnt2 shRNA1: GCAGTGACAATATTGACTACG

Wnt2 shRNA2: GCTGGAAGGAAGGCTGTAAAG

Wnt2 shRNA3: GCTTCACTGTAGCCAATAAGA

Wnt2 shRNA4: GCCTGTAGCCAAGGAGAATTA (effective)

**(2) DAP5 shRNA target sequences:**

DAP5 shRNA1: GCACTAGACGAGATGACAA

DAP5 shRNA2: GGAGTTATCCAGGATAGAT

DAP5 shRNA3: CCAGCTTCTTACAGTACAT (effective)

**(3) Primer information for real time RT-PCR:**

| Gene names | Forward primers | Reverse primers |
|---|---|---|
| *Gapdh* | TGGATTTGGACGCATTGGTC | TTTGCACTGGTACGTGTTGAT |

| | | |
|---|---|---|
| *Axin2* | AACTCAGTAACAGCCCAAGAAC | TCTCTCTTAAGTCAGCAGGG |
| *Wnt1* | CGCAGCACAGAACCAGCAAG | GCGGTCAGTGCCAGTAGCAA |
| *Wnt2* | CTCGGTGGAATCTGGCTCTG | CACATTGTCACACATCACCCT |
| *Wnt2b* | CAGACTTCCGACGCACAGGTGACTA | ATTGGCCCCATCCTGTGTGG |
| *Wnt3* | CTCGCTGGCTACCCAATTTG | CTTCACACCTTCTGCTACGCT |
| *Wnt3a* | CTCCTCTCGGATACCTCTTAGTG | GCATGATCTCCACGTAGTTCCTG |
| *Wnt4* | AGACGTGCGAGAAACTCAAAG | GGAACTGGTATTGGCACTCCT |
| *Wnt5a* | CAACTGGCAGGACTTTCTCAA | CATCTCCGATGCCGGAACT |
| *Wnt5b* | CTGCTGACTGACGCCAACT | CCTGATACAACTGACACAGCTTT |
| *Wnt6* | CCGGAAGTAGTGGCAGAGCTT | CCAGCGTCGGAAACGGAACT |
| *Wnt7a* | TGAACTTACACAATAACGAGGCG | GTGGTCCAGCACGTCTTAGT |
| *Wnt7b* | TTTGGCGTCCTCTACGTGAAG | CCCCGATCACAATGATGGCA |
| *Wnt8a* | GGGAACGGTGGAATTGTCCTG | GCAGAGCGGATGGCATGAA |
| *Wnt8b* | CCCGTGTGCGTTCTTCTAGTC | AGTAGACCAGGTAAGCCTTTGG |
| *Wnt9a* | GGCCCAAGCACACTACAAG | AGAAGAGATGGCGTAGAGGAAA |
| *Wnt9b* | CTGGTGCTCACCTGAAGCAG | CCGTCTCCTTAAAGCCTCTCTG |
| *Wnt10a* | CTGAGTTCTGGGCGCTCCTGTTCTT | TGTGTTGGCGTTGAGCACGG |
| *Wnt10b* | GAAGGGTAGTGGTGAGCAAGA | GGTTACAGCCACCCCATTCC |
| *Wnt16* | GCAGGCTGTCGCCAAGTTA | GTCTGCCTCTGGTCTTTTTCTC |

| | | |
|---|---|---|
| *Fzd1* | CAGCAGTACAACGGCGAAC | GTCCTCCTGATTCGTGTGGC |
| *Fzd2* | GCCGTCCTATCTCAGCTATAAGT | TCTCCTCTTGCGAGAAGAACATA |
| *Fzd3* | ATGGCTGTGAGCTGGATTGTC | GGCACATCCTCAAGGTTATAGGT |
| *Fzd4* | TGCCAGAACCTCGGCTACA | ATGAGCGGCGTGAAAGTTGT |
| *Fzd5* | AACAGCTGGAGTCTAGGCAT | CTCAGGCTTAACTCTCCCTA |
| *Fzd7* | GCCACACGAACCAAGAGGAC | CGGGTGCGTACATAGAGCATAA |
| *Fzd8* | ATGGAGTGGGGTTACCTGTTG | CACCGTGATCTCTTGGCAC |
| *Fzd9* | TTGCTCTATTATTTCGGGATGGC | CAGGACCACGATAGTTTTGAGTG |
| *Fzd10* | CATGCCCAACCTGATGGGTC | GCCACCTGAATTTGAACTGCTC |
| *Lrp6* | TTGTTGCTTTATGCAAACAGACG | GTTCGTTTAATGGCTTCTTCGC |

**(4) Information of primary antibodies used.**

| Antibody | Host Species | Dilution | Cat # | Registry ID | Source |
|---|---|---|---|---|---|
| GFAP | Rabbit | IHC 1:1000 | Z0334 | AB_10013382 | Dako |
| | Mouse | IHC 1:100 | AMAb91033 | AB_2665775 | Calbiochem |
| Nestin | Goat | IHC 1:200 | sc-23927 | AB_627994 | Santa Cruz |

| | | | | | |
|---|---|---|---|---|---|
| | Mouse | WB 1:1000 | GTX30671 | AB_625325 | GeneTex |
| NG2 | Rabbit | IHC 1:100 | AB5320 | AB_11213678 | Millipore |
| Sox2 | Rabbit | IHC 1:50 | #23064 | AB_2714146 | Cell Signaling |
| Pax6 | Rabbit | IHC 1:200 | 12323-1-AP | AB_2159695 | Proteintech |
| DCX | Rabbit | IHC | ab18723 | AB_732011 | Abcam |
| | Guinea pig | 1:400 | AB2253 | AB_1586992 | Millipore |
| | | IHC | | | |
| | | 1:1000 | | | |
| BrdU | Mouse | IHC | ab8152 | AB_308713 | Abcam |
| | Rat | 1:400 | | | |
| | | IHC | ab8955 | | Abcam |
| | | 1:400 | | | |
| GFP | Goat | IHC | 600-101-215 | AB_218182 | Rockland |
| | Rabbit | 1:500 | ab6556 | AB_305564 | Abcam |
| | | IHC | | | |
| | | 1:500 | | | |
| β-galactosidase | Rabbit | IHC 1:1000 | A-11132 | AB_221539 | MP |

| | | | | | |
|---|---|---|---|---|---|
| | Rabbit | WB 1:1000 | 0855976 | AB_2334934 | MP biomedical |
| | Mouse | WB 1:1000 | 2372 | AB_331784 | Cell Signaling |
| Wnt2 | Rabbit | IHC 1:50, WB 1:600 | 27214-1-AP | AB_2880804 | Proteintech |
| NeuN | Mouse | IHC 1:100 | MAB377 | AB_2298772 | Millipore |
| | Rabbit | IHC 1:1000 | ABN78 | AB_10807945 | Millipore |
| Axin2 | Rabbit | IHC 1:500 | ab109307 | AB_10862550 | Abcam |
| | Rabbit | WB 1:1000 | ab32197 | AB_2290204 | Proteintech |
| β-catenin | Rabbit | IHC 1:500, WB 1:600 | 06-734 | AB_310231 | Millipore |
| Cleaved Caspase -3 | Rabbit | WB 1:1000 | 9664S | AB_2070042 | Cell Signaling Technology |

| | | | | | |
|---|---|---|---|---|---|
| | | 1：400 | 9961 | AB_2341188 | Cell Signaling Technology |
| Caspase-3 | Rabbit | WB | 9962 | AB_331439 | Cell Signaling Technology |
| BSA | Rabbit | WB 1:500 | 66201-1-Ig | AB_2881592 | Proteintech |
| LMNB1 | Mouse | WB 1:800 | 12987-1-AP | AB_2136290 | Proteintech |
| β-actin | Mouse | WB 1:5000 | A5441 | AB_476744 | Sigma |
| Ki67 | Rabbit | IHC | ab15580 | AB_443209 | Abcam |
| Active | Rabbit | 1:200 | 8814 | AB_11127203 | Cell Signaling |
| β-catenin | Mouse | WB 1:1000 | 66379-1-Ig | AB_2857358 | Proteintech |
| ALDH1L1 | Mouse | IHC 1: 200 | 68018-1-Ig | Not registered | Proteintech |
| DAP5 | Mouse | WB 1: 100 | sc-137011 | AB_2095908 | Santa Cruz Biotech |
| Ngn2 | Rabbit | 1:100 | GTX129258 | AB_2885944 | GeneTex |

## Supplementary figure legends:

**Supplementary Fig. 1. Expression of neural progenitor marker and proliferation of reactive astrocytes in ischemic cortex.**

(a) Double-immunostaining and quantification of Nestin/NG2 in the ischemic region. (b) Double-immunostaining and quantification of Nestin/Iba-1 in the ischemic region. (c) Double-immunostaining and quantification of Sox2/NG2 in the ischemic region. (d) Double-immunostaining and quantification of Sox2/Iba-1 in the ischemic region. (e, f) Triple-immunostaining and quantification of GFAP/BrdU/Nestin and GFAPBrdU/Sox2 in the ischemic region. Bars = 50 μm.

**Supplementary Fig. 2. Expression of Nestin in local reactive astrocytes.**

Double-immunostaining of GFAP/Nestin in Nestin-CreER:ROSA-DTA mice at 7 dpi pre-treated with TAM or oil (control). Notice the depletion of Nestin-positive cells in SVZ and the GFAP/Nestin-positive cells in the ischemic cortex of mice pretreated with TAM (right panels).

**Supplementary Fig. 3. Expression of Wnt ligands in normal and ischemic mouse cortex.**

(a) Real time RT-PCR of Wnt ligands in the normal adult cortex. Notice that Wnt2, Wnt4, Wnt7a, Wnt9a and Wnt10a show relatively higher expression level (N=3 mice). (b) In situ hybridization of Wnt7a, Wn9a, Wnt10a and Wnt2 in the intact cortex. Bar = 50 μm. (c) Double-immunostaining of GFAP/Wnt2 in normal cortex. Bars = 50 μm. (d) Efficacy of Wnt2 shRNA in silencing Wnt2 mRNA expression *in vivo.* *$P$=0.021. Two-tailed Student's

t-test. N = 4 mice per group. (e) Efficacy of Wnt2 shRNA in reducing Wnt2 protein expression *in vivo*. (f) Western-blotting of Wnt4 at different time points after ischemia. (g) Western-blotting of Wnt7a at different time points after ischemia. (h) Western-blotting of Wnt9a at different time points after ischemia. (i) Western-blotting of Wnt10a at different time points after ischemia. Notice that there was no significant change of Wnt4, Wnt7a, Wnt9a and Wnt10a in the ischemic cortex. (j) Western-blotting of Wnt2 and cleaved caspase-3 (CC-3) in primary neurons at different time points after OGD-treatment. Notice that pan-caspase inhibitor z-VAD blocked the up-regulation of Wnt2 and CC-3. $^{**}P_{\text{Wnt2-2h}} = 0.0018$, $^{**}P_{\text{Wnt2-6h}} = 0.0031$, $^{**}P_{\text{Wnt2-12h}} = 0.0033$, $^{**}P_{\text{Wnt2-24h}} = 0.0029$, $^{*}P_{\text{Wnt2-24h vs Wnt2-24h+z-VAD}} = 0.018$, $^{**}P_{\text{CC3-2h}} = 0.0023$, $^{**}P_{\text{CC3-6h}} = 0.0020$, $^{**}P_{\text{CC3-12h}} = 0.0021$, $^{**}P_{\text{CC3-24h}} = 0.0026$, $^{*}P_{\text{CC3-24h vs CC3+z-VAD}} = 0.039$. One-way ANOVA followed by Bonferroni's post hoc comparisons tests. N = 3 batches of cells. Asterisks indicate the comparison with control group. Asterisks with bars connecting two groups indicate difference between these two groups. (k) Western-blotting of cleaved Caspase-3 in primary astrocytes treated with or without OGD. $^{***}P < 0.001$. One-way ANOVA followed by Bonferroni's post hoc comparisons tests. N = 3 batches of cells. (l) ELISA of Wnt2 in conditioned medium of control neurons, OGD-treated neurons, and OGD plus Wnt2 shRNA treated neurons. $^{***}P_{\text{Con-OGD}} < 0.001$, $^{**}P_{\text{OGD-OGD Wnt2 shRNA}} = 0.002$. One-way ANOVA followed by Bonferroni's post hoc comparisons tests. N = 3 batches of cells. (m) Western-blotting of caspase-3 in control neurons and Wnt2 shRNA treated neurons. Two-tailed Student's t-test. N = 3 batches of cells. Mean ± standard error (SE).

**Supplementary Fig. 4. The cell types lentivirus infected.**

(a) Double-immunostaining of GFP with GFAP, NeuN, NG2 and Iba-1 in lenti-GFP infected cortex. Bar = 50 μm. (b) Quantification of different cell types infected by lenti-GFP.

**Supplementary Fig. 5. Effects of Wnt2/TCF4 manipulation on astrocyte dedifferentiation.**

(a) Double-immunostaining of GFAP/Ngn2 in normal cortex (Contra), ischemic cortex (Ipsi) and ischemic cortex pre-treated with Wnt2 shRNA. (b) Western-blotting of β-gal and Nestin in ischemic cortex of Topgal mice infected by pLenti-Luci (control) or dnTCF-4. Notice that dnTCF-4 reduces the expression of β-gal and Nestin in the ischemic cortex. *$P$<0.05, **$P$<0.01. Two-tailed Student's $t$ test. N = 3 mice per group. (c) Double-immunostaining and quantification of DCX/BrdU in ischemic cortex of infected by pLenti-Luci (control) and dnTCF-4. N = 3 mice per group. ***$P$<0.001. Two-tailed Student's $t$ test. Bar =50 μm. (d-f) Double-immunostaining of Aldh1l1/Nestin and Western-blotting of Nestin in primary astrocytes treated with Wnt2 protein. Notice the up-regulation of Nestin. *$P$<0.05, **$P$<0.01. N = 3 batches of cells. Two-tailed Student's $t$ test. (g) Double-immunostaining and quantification of Nestin/CD31 in control (Con), Wnt2-shRNA and Lenti-Wnt2 treated ischemic cortex. (h) Double-immunostaining and quantification of CD31/Ki67 in control (Con), Wnt2-shRNA and Lenti-Wnt2 treated ischemic cortex. Mean ± standard error (SE).

**Supplementary Fig. 6. Effects of caspase-3 knockout on the expression of Axin2 in cortex and proliferation of NSCs in SVZ.**

(a) Western-blotting and quantification of Axin2 in the ischemic cortex of wild type (WT) and Caspase-3$^{-/-}$ mice at 5 dpi. Expression of Axin2 in the injured area of Caspase-3$^{-/-}$ cortex is

much lower than that in the injured area of wild type mice. ***P*=0.0014. One-way ANOVA followed by Bonferroni's post hoc comparisons tests. N = 3-4 mice per group. (b) Combination of TUNEL staining with immunostaining of NeuN in WT and Caspase-3$^{-/-}$ cortex at 24 h post ischemia. Images in the frame was enlarged for showing typical double-stained cells. N = 3 mice per group. ***P*<0.01. Two-tailed Student's t-test. (c) Double-immunostaining of Nestin/Ki67 in the SVZ of WT and Caspase-3$^{-/-}$ mice. N = 3 mice per group. Two-tailed Student's t-test. Mean ± standard error (SE).

**Supplementary Fig. 7. Effects of EbC on cell survival in Caspase-3$^{-/-}$ mice.**

Combination of TUNEL staining with immunostaining of DCX or NeuN in Caspase-3$^{-/-}$ cortex treated with EbC or Lenti-Luci (as control). No significant difference of the quantification of TUNEL/DCX- and TUNEL/NeuN-positive cells was found. N = 3 mice per group. Two-tailed Student's t-test. Mean ± standard error (SE).

**Supplementary Fig. 8. Neuronal apoptosis in** Macaca Mulatta **and human patients at 24 h post ischemia.**

Double-immunostaining of NeuN/CC-3 in Macaca Mulatta and human cortex at 24 h post ischemia. Arrows point to double-stained cells. Bars = 20 μm.

**Supplementary Fig. 9. Involvement of DAP5 in Wnt2 up-regulation and astrocyte dedifferentiation.**

(a) Real time RT-PCR of Wnt2 in cortex after ischemia. Wnt2 mRNA remains unchanged *in vivo*. (b) Western-blotting of Wnt2 in MCF7 cells after OGD treatment. ***P*<0.01. N=3 batches of cells. Two-tailed Student's t-test. (c) Western-blotting of DAP5 in the contralateral (Contra) and ipsilateral (Ipsi) cortex at 24 h post ischemia. **P*<0.05. N=3 batches of cells. Two-tailed Student's t-test. (d) Western-blotting of DAP5 in neurons treated by different shRNA targeting DAP5. (e) Western-blotting of Wnt2 in normal and OGD-treated neurons, which were treated by either control shRNA or DAP5 shRNA. Notice that DAP5 shRNA significantly attenuated the OGD up-regulated Wnt2 protein. **P*<0.05. One-way ANOVA followed by Bonferroni's post hoc comparisons tests. N = 3 batches of cells. (f) Double-immunostaining of GFAP/Sox2 in ischemic cortex treated by control and DAP5 shRNA. There were less Sox2-positive astrocytes in DAP5 shRNA treated cortex. Mean ± standard error (SE).

# Suppl.Fig-1

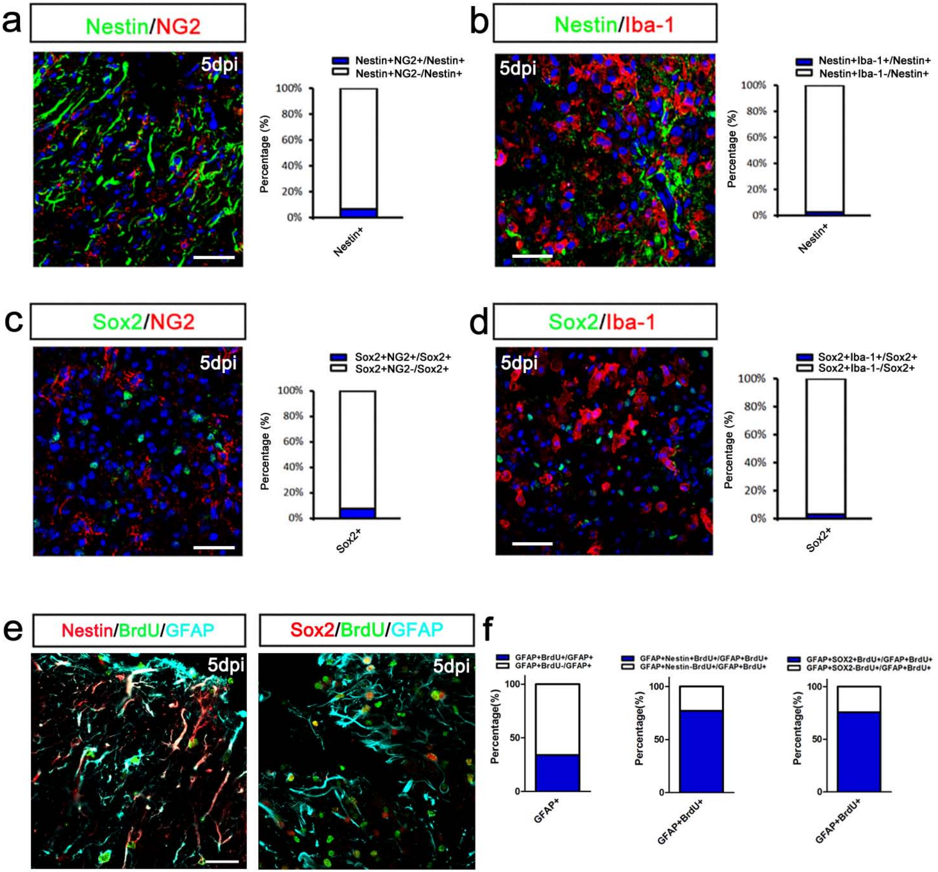
a
Nestin/NG2
5dpi
Nestin+NG2+/Nestin+
Nestin+NG2-/Nestin+
Percentage (%)
Nestin+
b
Nestin/Iba-1
5dpi
Nestin+Iba-1+/Nestin+
Nestin+Iba-1-/Nestin+
Percentage (%)
Nestin+
c
Sox2/NG2
5dpi
Sox2+NG2+/Sox2+
Sox2+NG2-/Sox2+
Percentage (%)
Sox2+
d
Sox2/Iba-1
5dpi
Sox2+Iba-1+/Sox2+
Sox2+Iba-1-/Sox2+
Percentage (%)
Sox2+
e
Nestin/BrdU/GFAP
5dpi
Sox2/BrdU/GFAP
5dpi
f
GFAP+BrdU+/GFAP+
GFAP+BrdU-/GFAP+
Percentage(%)
GFAP+
GFAP+Nestin+BrdU+/GFAP+BrdU+
GFAP+Nestin-BrdU+/GFAP+BrdU+
Percentage(%)
GFAP+BrdU+
GFAP+SOX2+BrdU+/GFAP+BrdU+
GFAP+SOX2-BrdU+/GFAP+BrdU+
Percentage(%)
GFAP+BrdU+

# Suppl. Fig-2

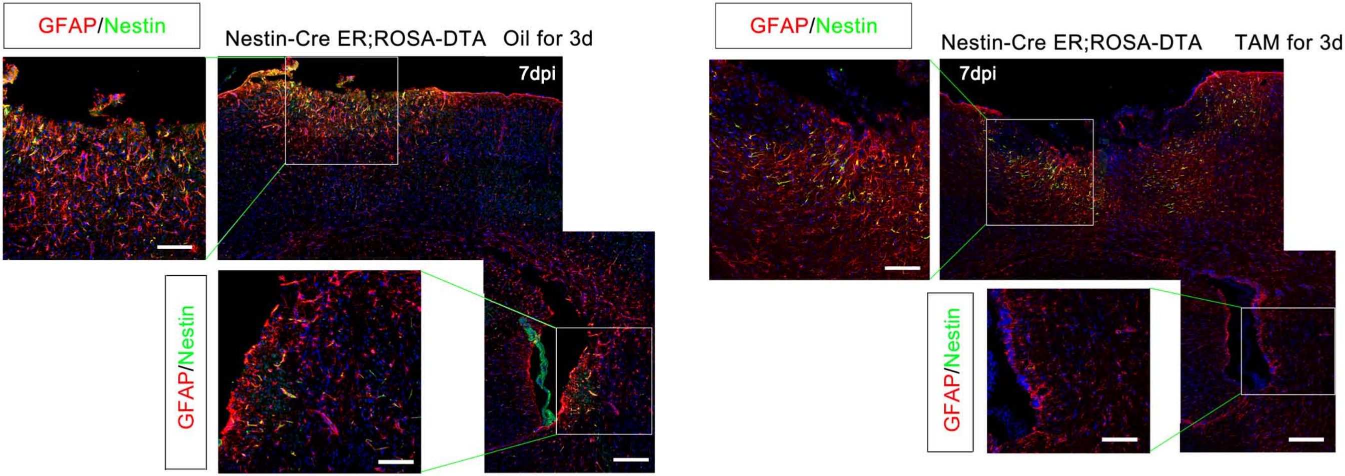
GFAP/Nestin
Nestin-Cre ER;ROSA-DTA Oil for 3d
7dpi
GFAP/Nestin
GFAP/Nestin
Nestin-Cre ER;ROSA-DTA TAM for 3d
7dpi
GFAP/Nestin

Suppl. Fig-3

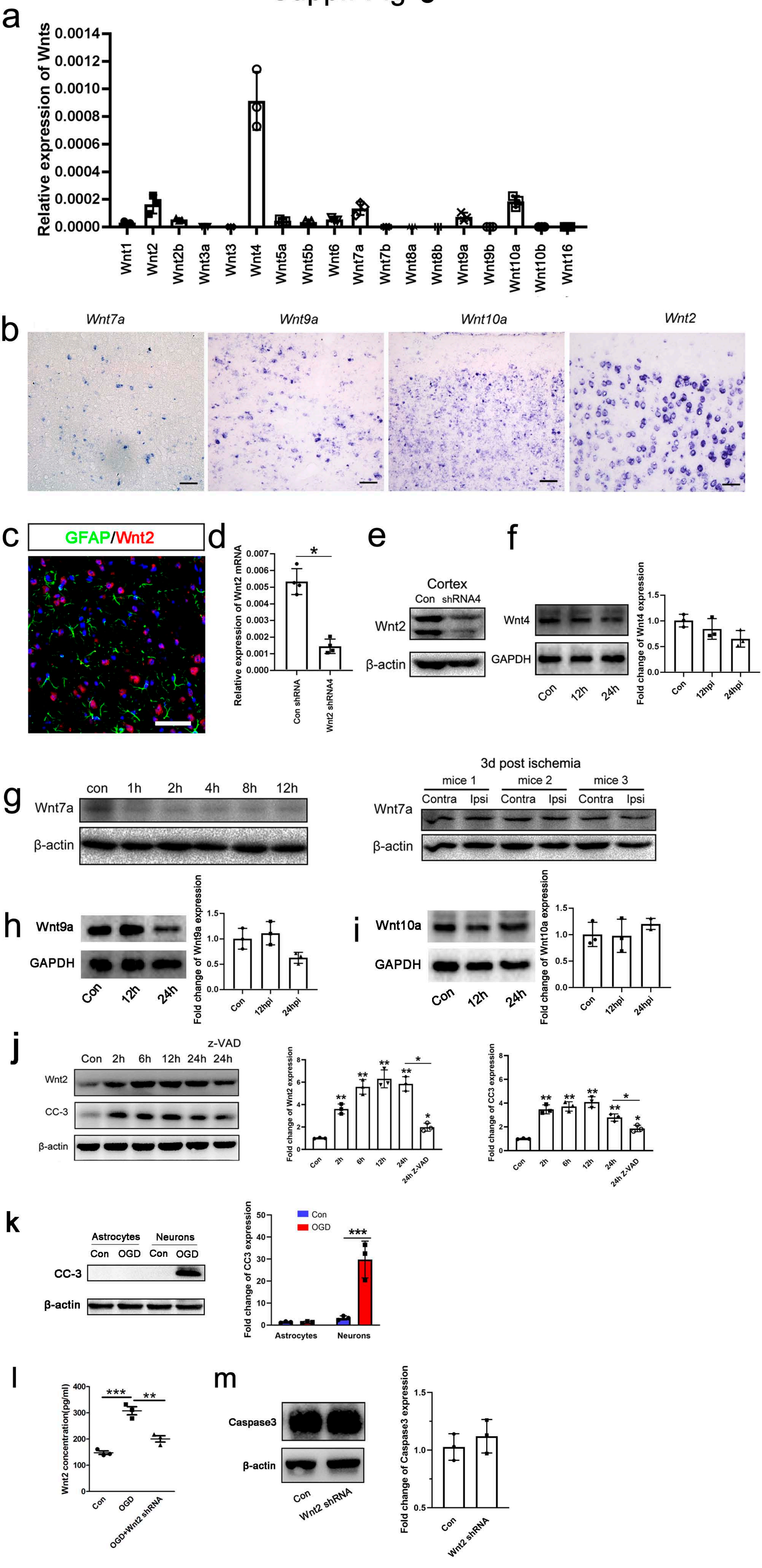
a
Relative expression of Wnts
0.0014
0.0012
0.0010
0.0008
0.0006
0.0004
0.0002
0.0000
Wnt1 Wnt2 Wnt2b Wnt3a Wnt3 Wnt4 Wnt5a Wnt5b Wnt6 Wnt7a Wnt7b Wnt8a Wnt8b Wnt9a Wnt9b Wnt10a Wnt10b Wnt16
b
Wnt7a
Wnt9a
Wnt10a
Wnt2
c
GFAP/Wnt2
d
Relative expression of Wnt2 mRNA
Con shRNA
Wnt2 shRNA4
e
Cortex
Con shRNA4
Wnt2
β-actin
f
Wnt4
GAPDH
Con 12h 24h
Fold change of Wnt4 expression
Con 12hpi 24hpi
g
con 1h 2h 4h 8h 12h
Wnt7a
β-actin
3d post ischemia
mice 1 mice 2 mice 3
Contra Ipsi Contra Ipsi Contra Ipsi
Wnt7a
β-actin
h
Wnt9a
GAPDH
Con 12h 24h
Fold change of Wnt9a expression
Con 12hpi 24hpi
i
Wnt10a
GAPDH
Con 12h 24h
Fold change of Wnt10a expression
Con 12hpi 24hpi
j
Con 2h 6h 12h 24h 24h
z-VAD
Wnt2
CC-3
β-actin
Fold change of Wnt2 expression
Con 2h 6h 12h 24h 24h Z-VAD
Fold change of CC3 expression
Con 2h 6h 12h 24h 24h Z-VAD
k
Astrocytes Neurons
Con OGD Con OGD
CC-3
β-actin
Fold change of CC3 expression
Con
OGD
Astrocytes Neurons
l
Wnt2 concentration(pg/ml)
Con OGD OGD+Wnt2 shRNA
m
Caspase3
β-actin
Con Wnt2 shRNA
Fold change of Caspase3 expression
Con Wnt2 shRNA

# Suppl. Fig-4

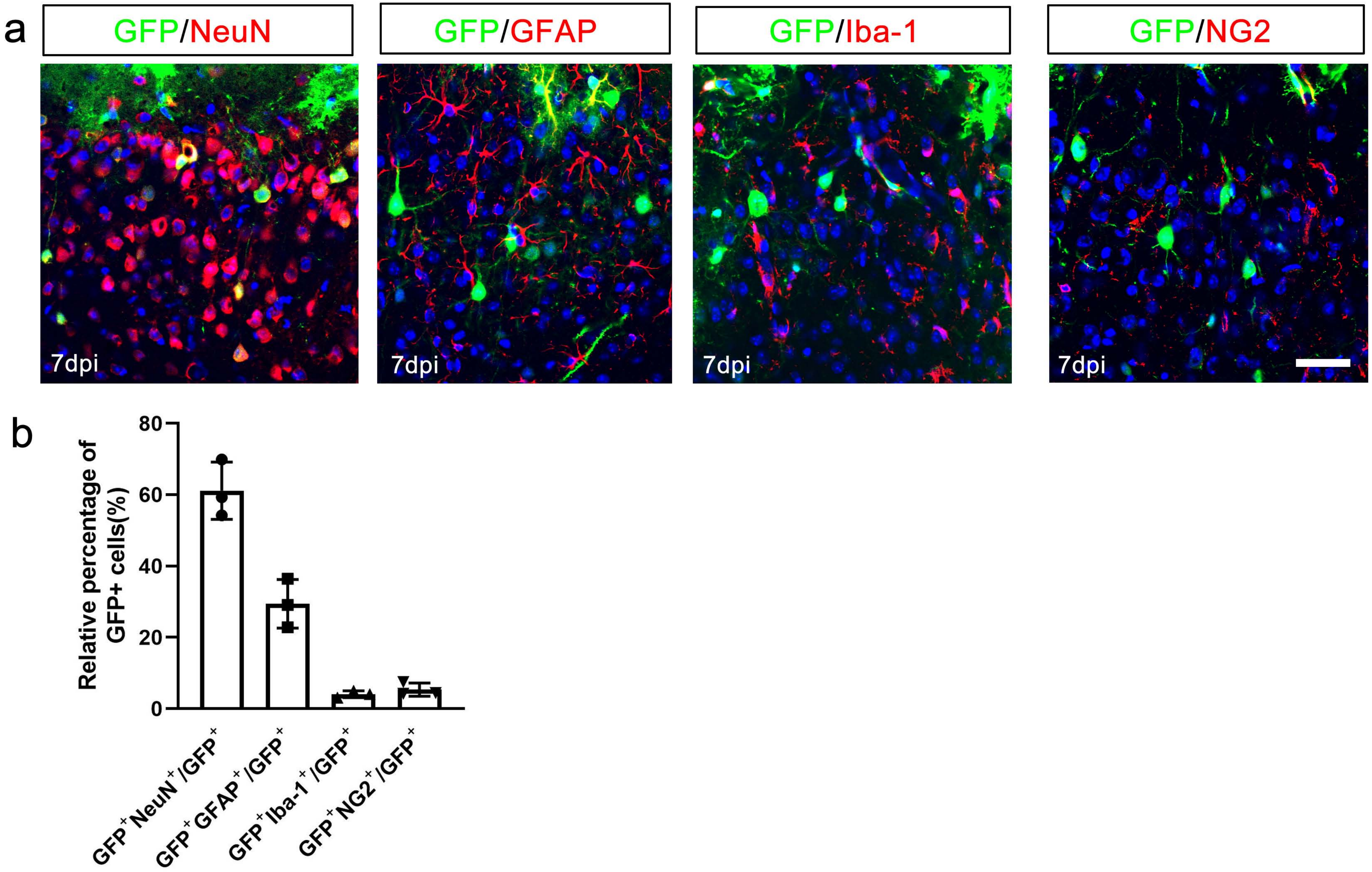
a
GFP/NeuN
GFP/GFAP
GFP/Iba-1
GFP/NG2
7dpi
7dpi
7dpi
7dpi
b
Relative percentage of GFP+ cells(%)
80
60
40
20
0
GFP+NeuN+/GFP+
GFP+GFAP+/GFP+
GFP+Iba-1+/GFP+
GFP+NG2+/GFP+

# Suppl.Fig-5

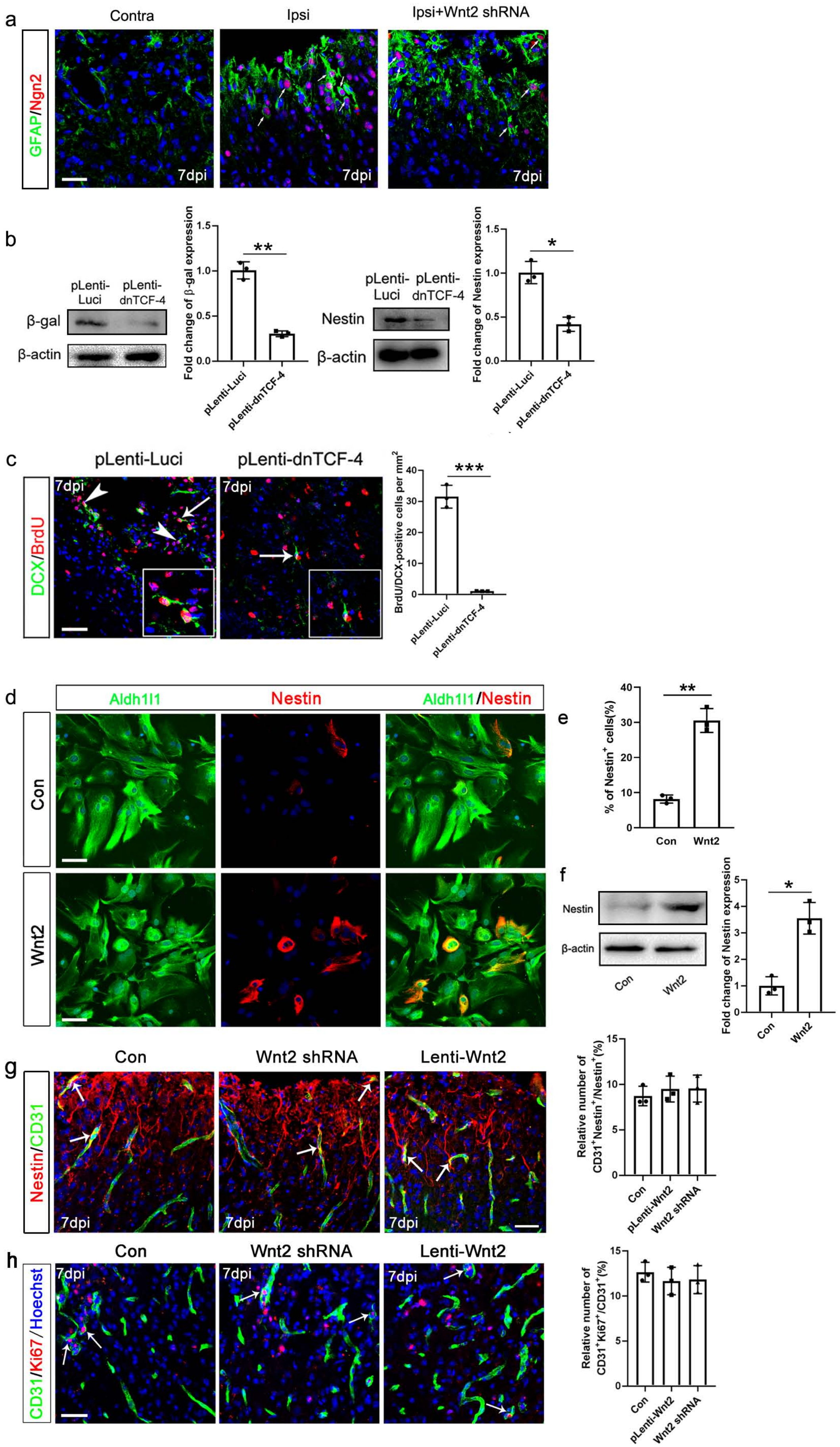

a
Contra
Ipsi
Ipsi+Wnt2 shRNA
GFAP/Ngn2
7dpi
b
pLenti-Luci
pLenti-dnTCF-4
β-gal
β-actin
Fold change of β-gal expression
**
Nestin
Fold change of Nestin expression
*
c
DCX/BrdU
pLenti-Luci
pLenti-dnTCF-4
BrdU/DCX-positive cells per mm²
***
d
Aldh1l1
Nestin
Aldh1l1/Nestin
Con
Wnt2
e
% of Nestin⁺ cells(%)
**
f
Nestin
β-actin
Con
Wnt2
Fold change of Nestin expression
*
g
Con
Wnt2 shRNA
Lenti-Wnt2
Nestin/CD31
Relative number of CD31⁺Nestin⁺/Nestin⁺(%)
pLenti-Wnt2
h
CD31/Ki67/Hoechst
Relative number of CD31⁺Ki67⁺/CD31⁺(%)

# Suppl.Fig-6

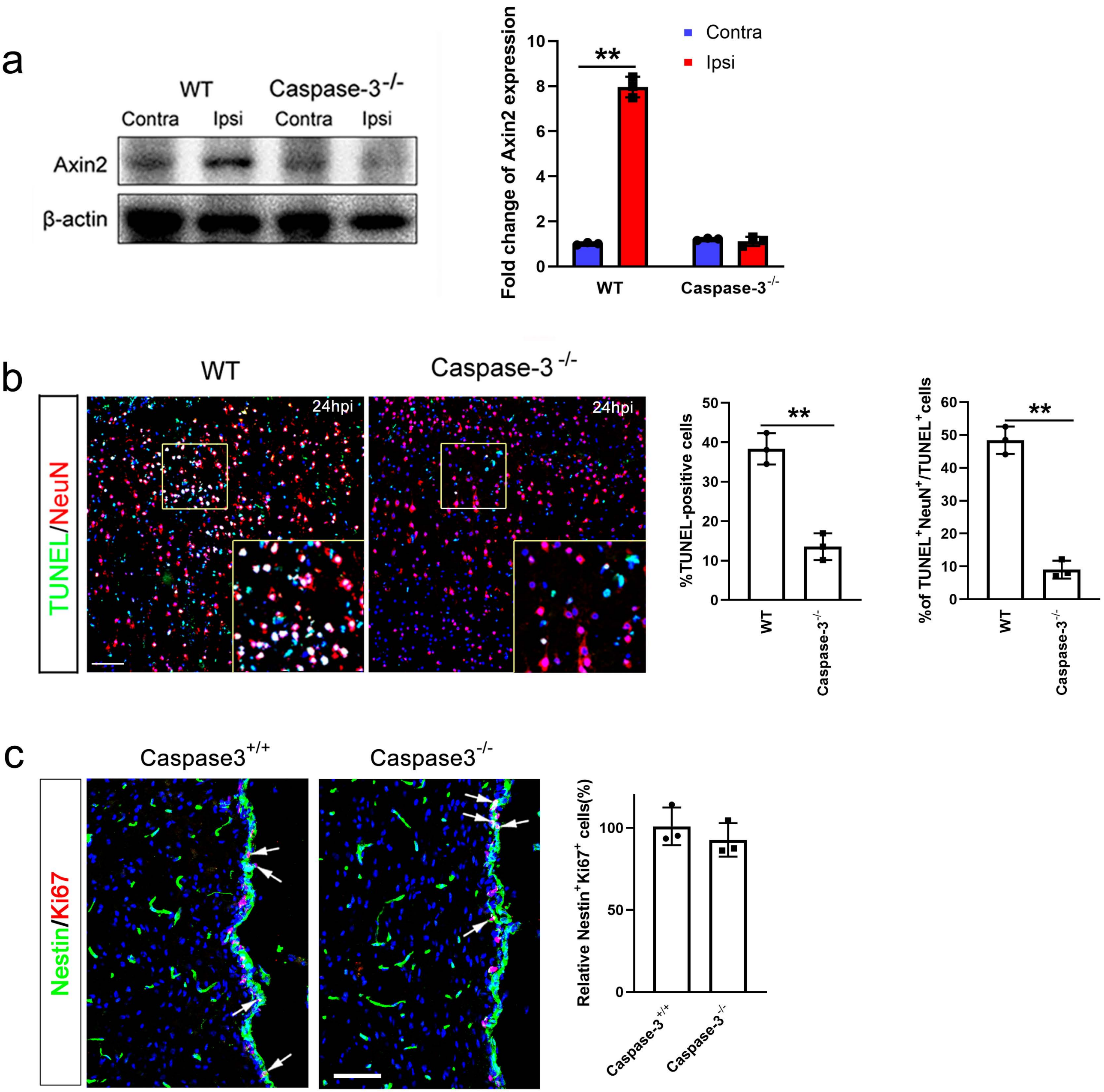

a
WT
Caspase-3-/-
Contra
Ipsi
Contra
Ipsi
Axin2
β-actin
Fold change of Axin2 expression
Contra
Ipsi
**
WT
Caspase-3-/-
b
WT
Caspase-3 -/-
TUNEL/NeuN
24hpi
24hpi
%TUNEL-positive cells
%of TUNEL+NeuN+/TUNEL+cells
**
**
WT
Caspase-3-/-
c
Caspase3+/+
Caspase3-/-
Nestin/Ki67
Relative Nestin+Ki67+ cells(%)
Caspase-3+/+
Caspase-3-/-

# Suppl. Fig-7

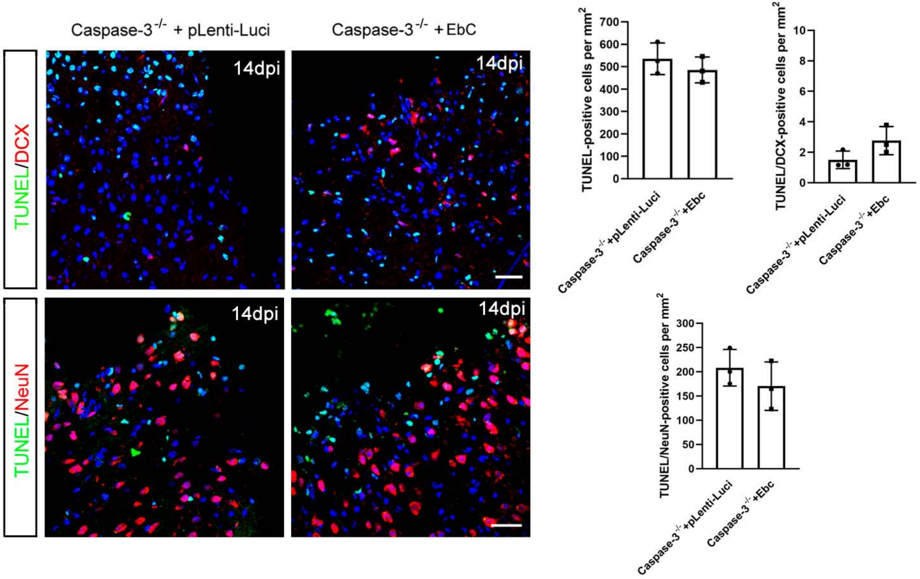
Caspase-3 -/- + pLenti-Luci
Caspase-3 -/- + EbC
TUNEL/DCX
TUNEL/NeuN
14dpi
TUNEL-positive cells per mm2
TUNEL/DCX-positive cells per mm2
TUNEL/NeuN-positive cells per mm2
Caspase-3 -/- +pLenti-Luci
Caspase-3 -/- +Ebc

# Suppl.Fig-8

Monkey 24hpi

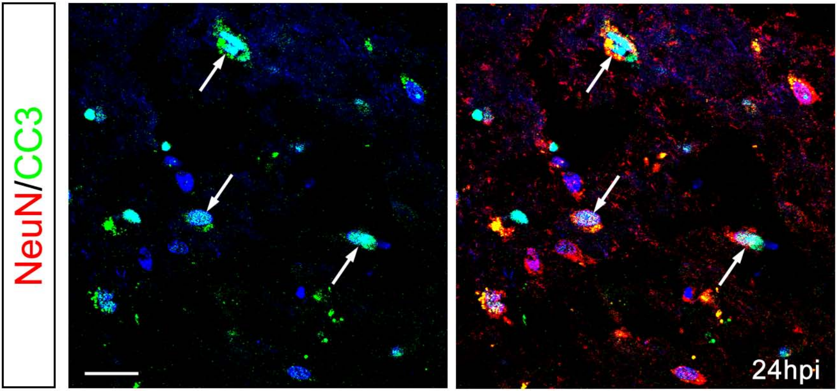


Human 24hpi

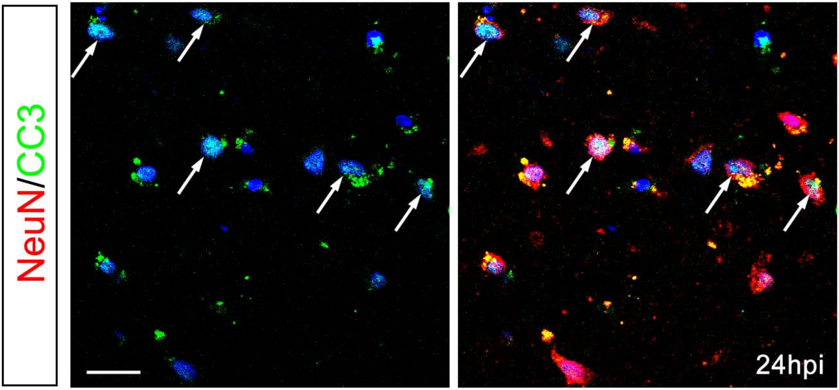

# Suppl. Fig-9

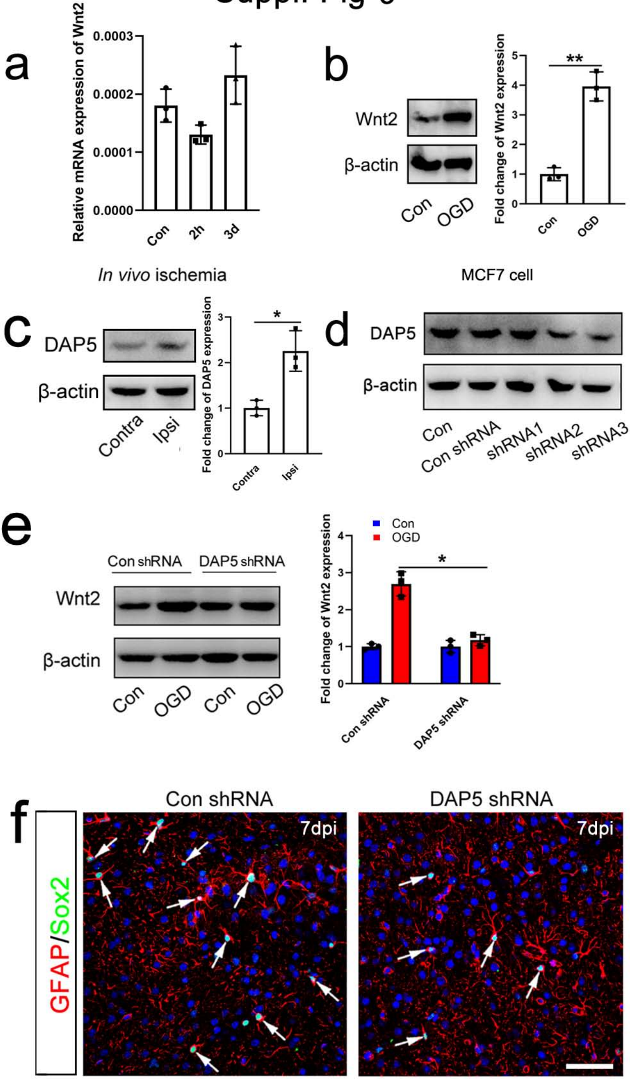
a
Relative mRNA expression of Wnt2
0.0003
0.0002
0.0001
0.0000
Con
2h
3d
In vivo ischemia
b
Wnt2
β-actin
Con
OGD
Fold change of Wnt2 expression
**
c
DAP5
β-actin
Contra
Ipsi
Fold change of DAP5 expression
*
d
MCF7 cell
DAP5
β-actin
Con
Con shRNA
shRNA1
shRNA2
shRNA3
e
Con shRNA
DAP5 shRNA
Wnt2
β-actin
Con
OGD
Con
OGD
Fold change of Wnt2 expression
*
f
Con shRNA
DAP5 shRNA
7dpi
7dpi
GFAP/Sox2

# Full gels of the main Figures (biological repeat-1)

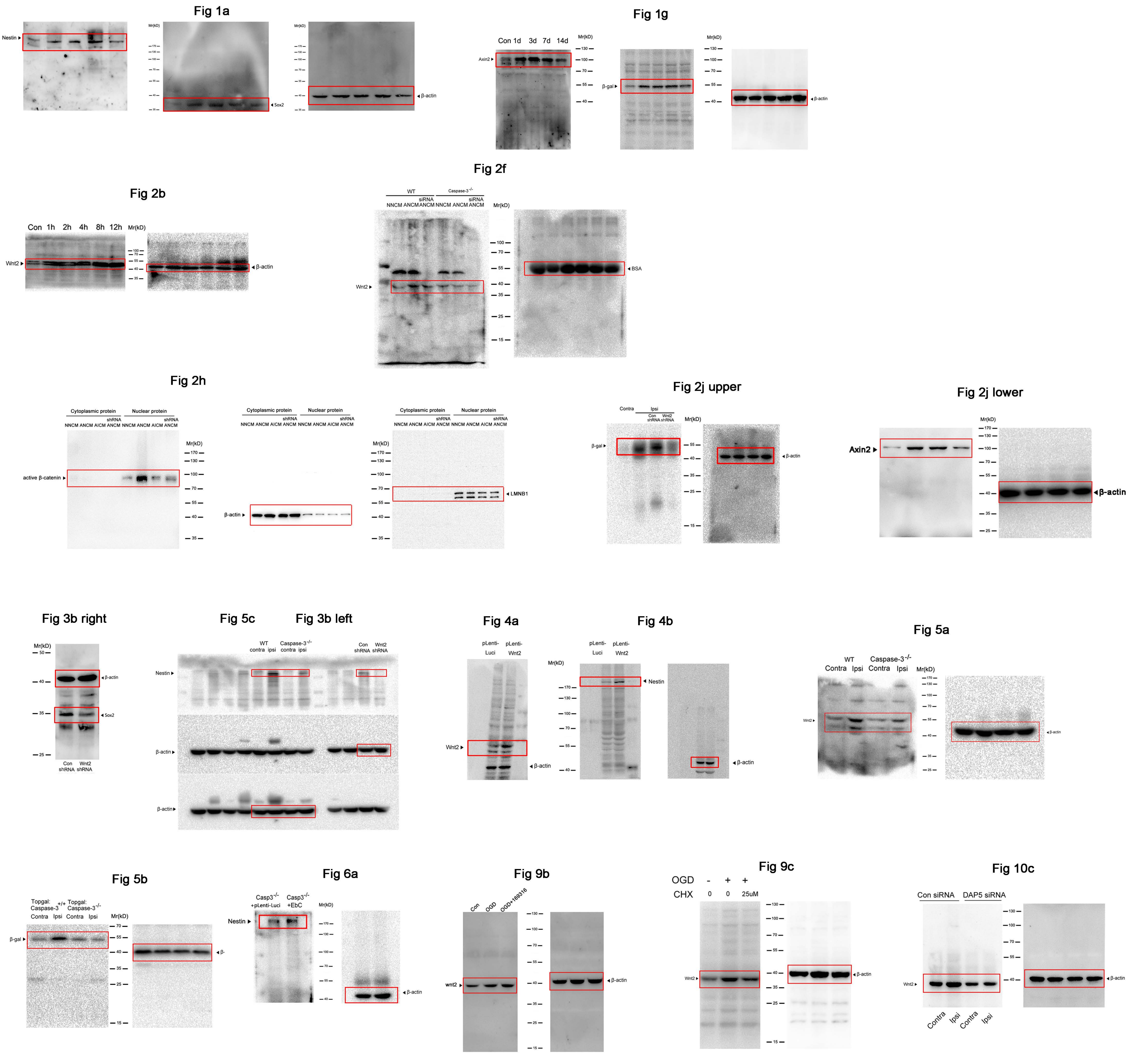

# Full gels of main Figures (biological repeat-2)

## Fig 1a

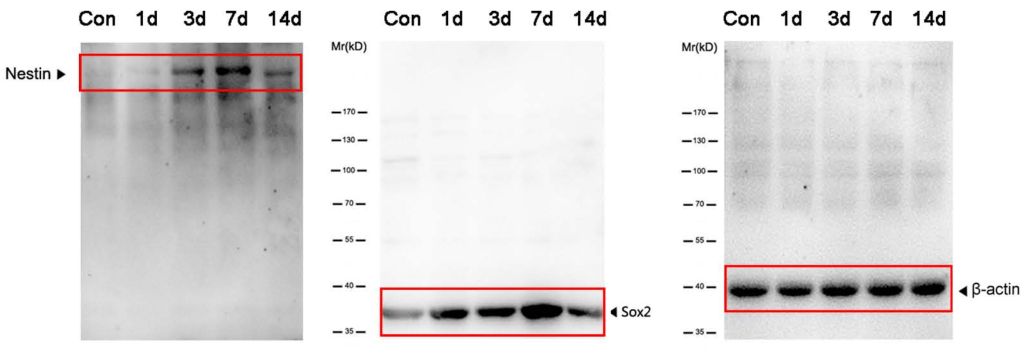


## Fig 1g

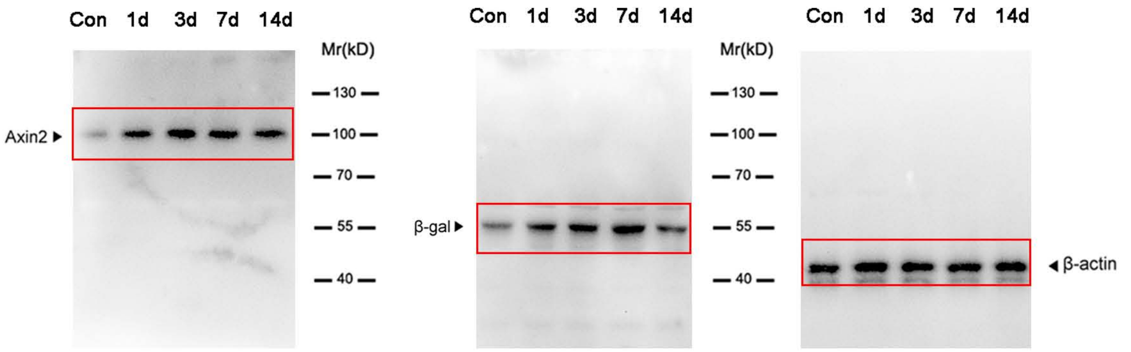


## Fig 2b

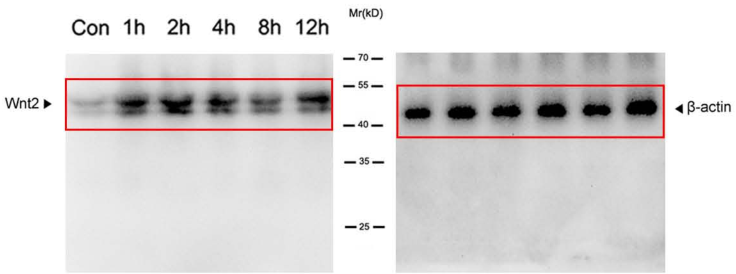


## Fig 2f

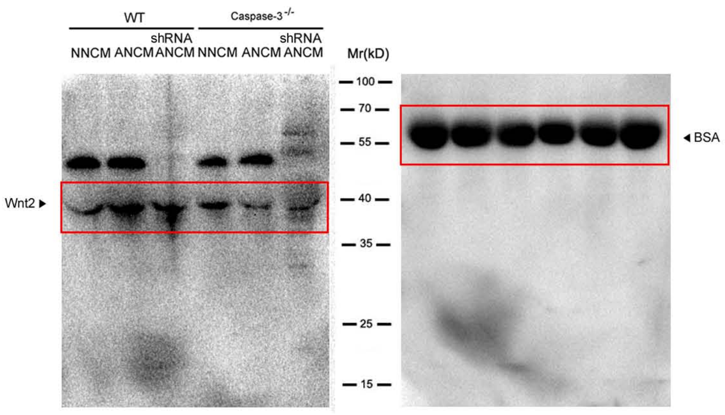


## Fig 2h

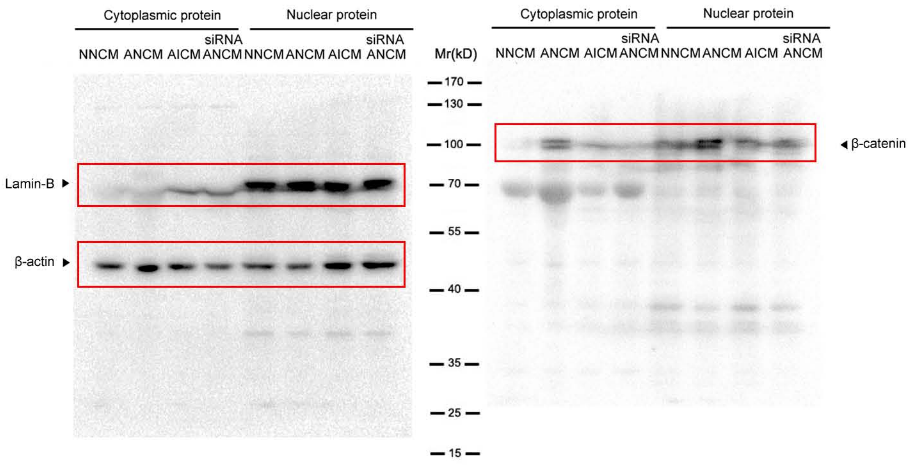


## Fig 2j upper

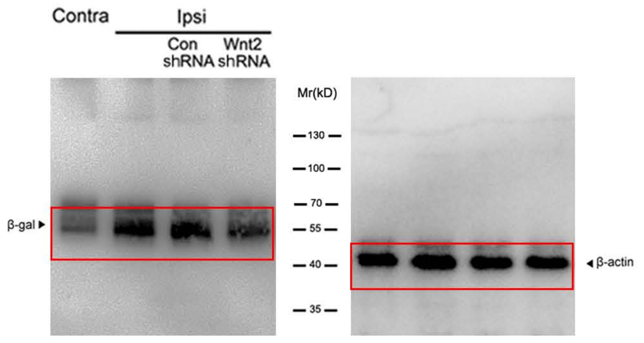


## Fig 2j lower

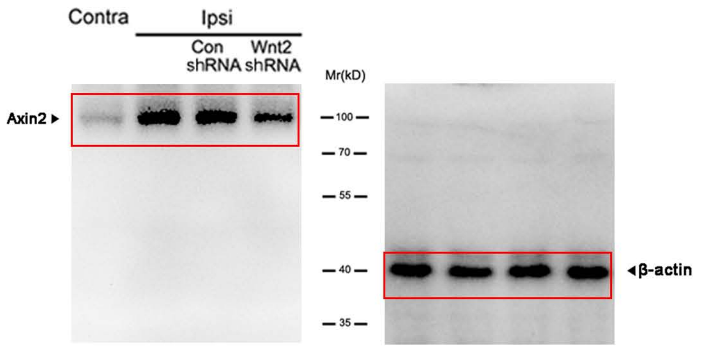


## Fig 3b left

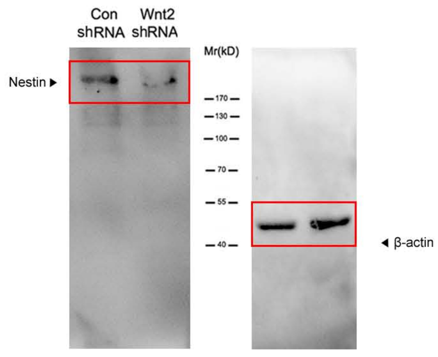


## Fig 3b right

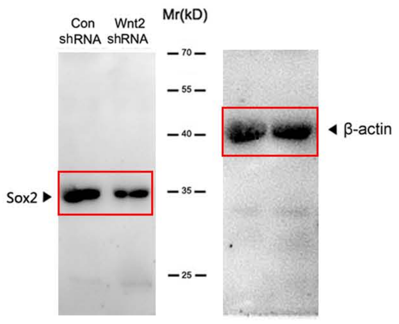


## Fig 4a

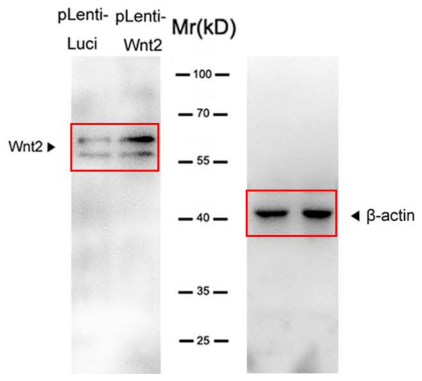


## Fig 4b

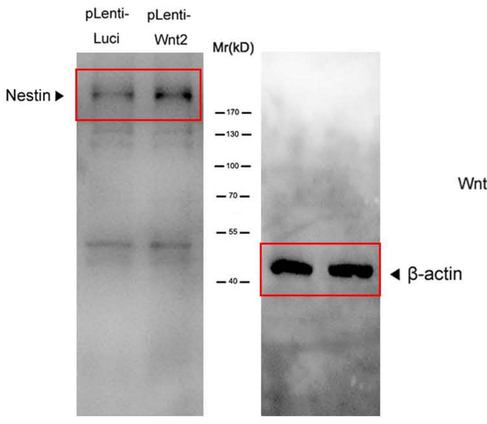


## Fig 5a

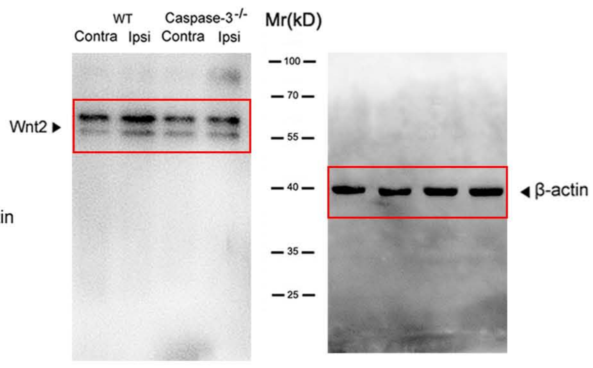


## Fig 5b

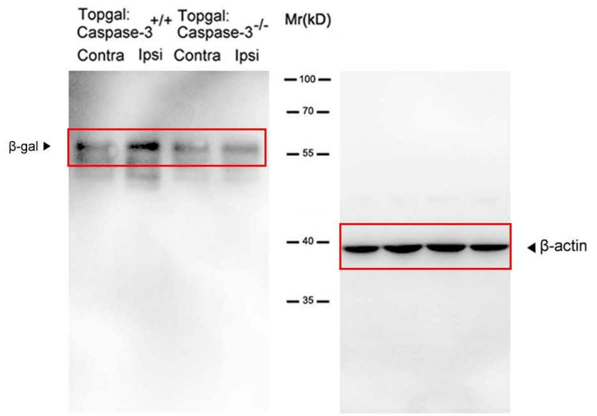


## Fig 5c

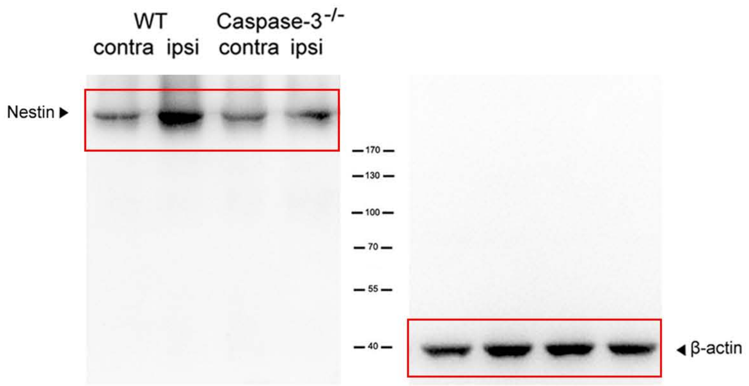


## Fig 6a

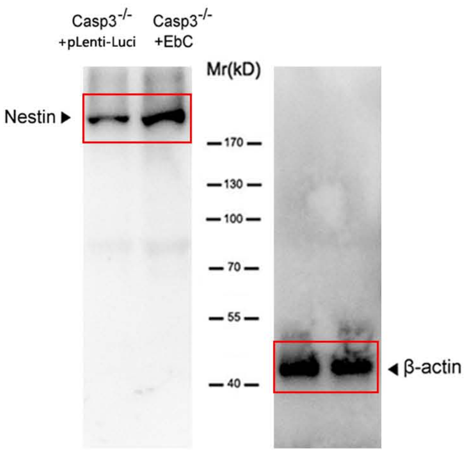


## Fig 9b

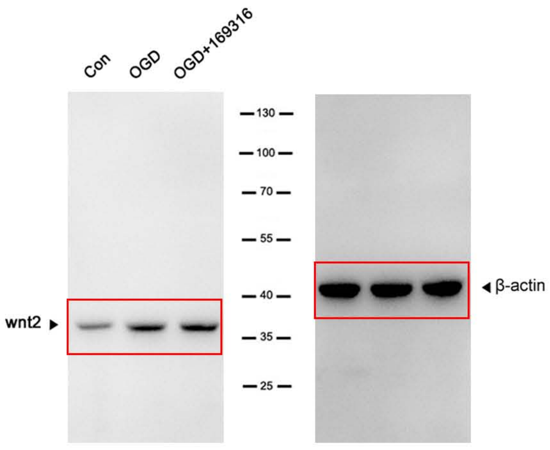


## Fig 9c

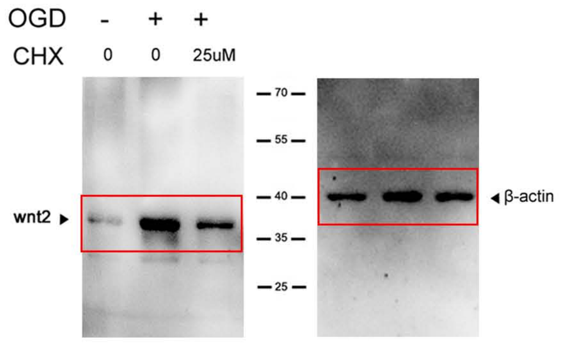


## Fig 10c

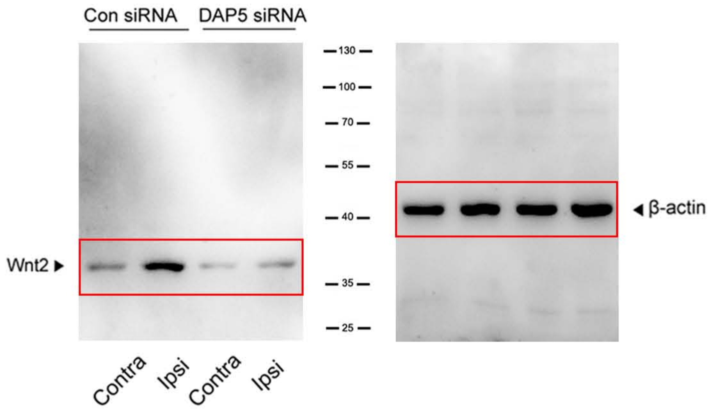

# Full gels of main Figures (biological repeat-3)

## Fig 1a

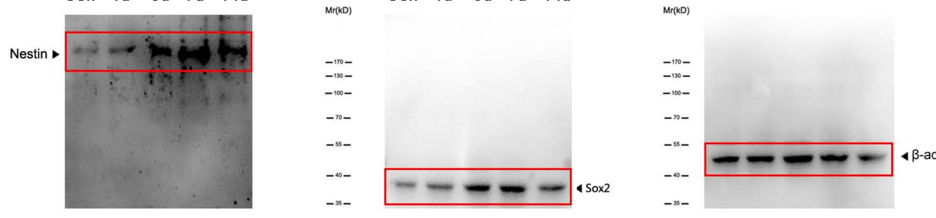


## Fig1g

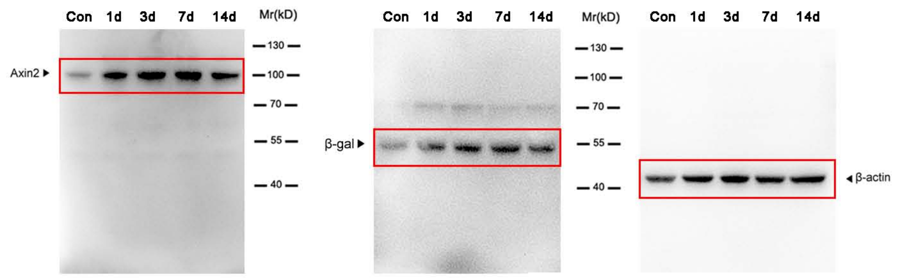


## Fig 2b

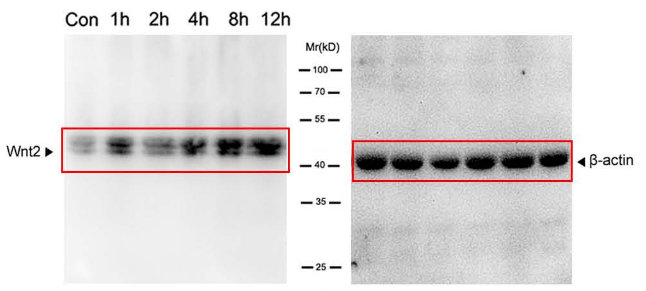


## Fig 2f

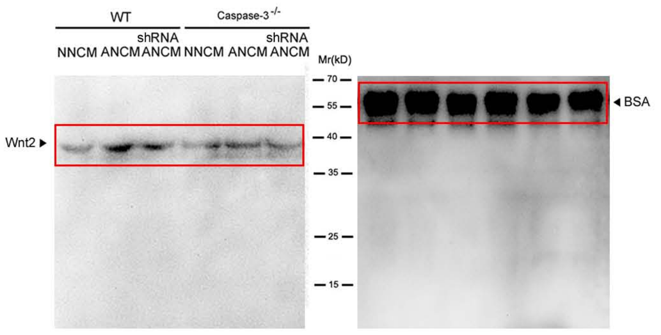


## Fig 2h

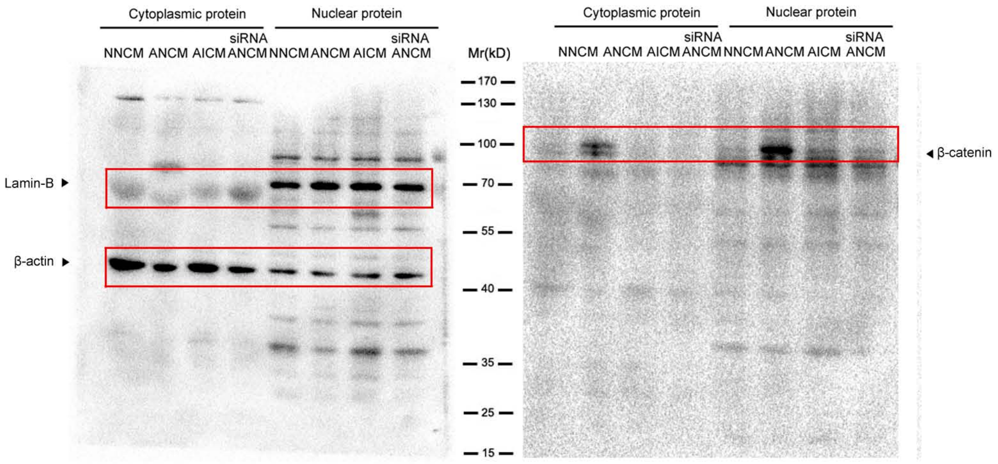


## Fig 2j upper

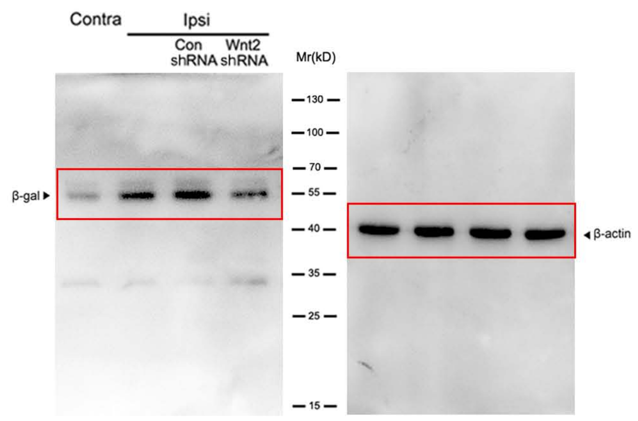


## Fig 2j lower

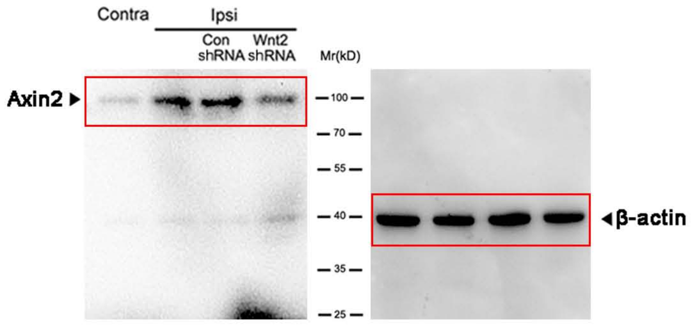


## Fig 3b left

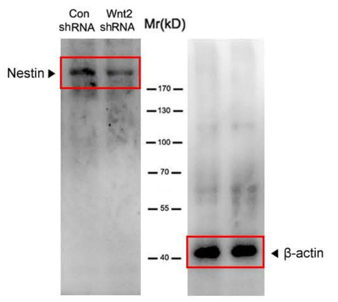


## Fig 3b right

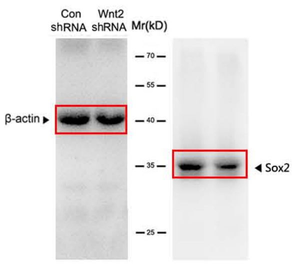


## Fig 4a

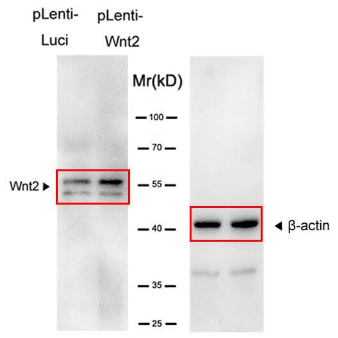


## Fig 4b

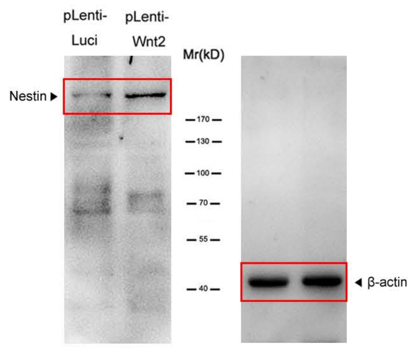


## Fig 5a

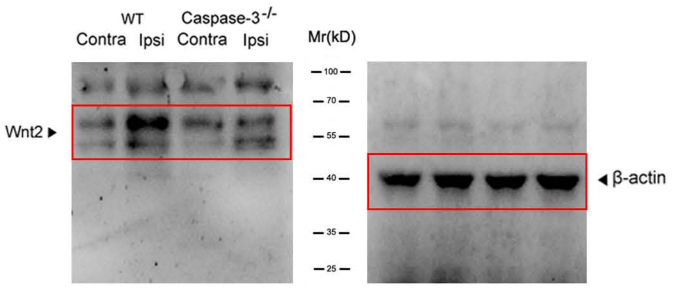


## Fig 5b

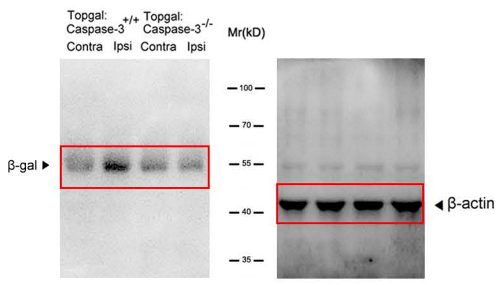


## Fig 5c

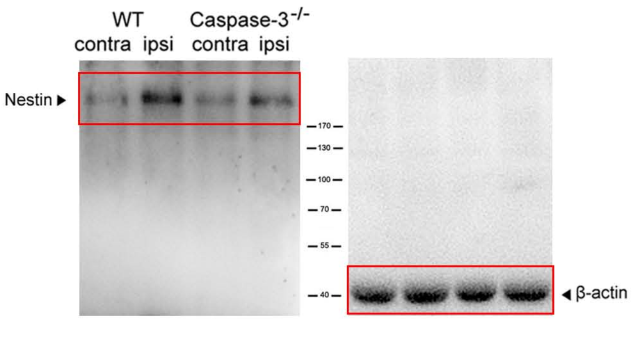


## Fig 6a

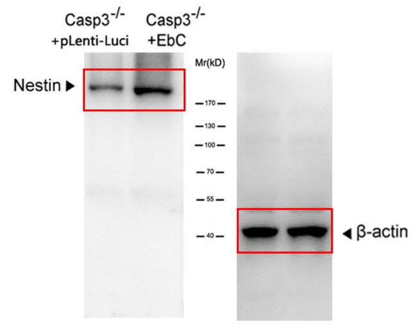


## Fig 9b

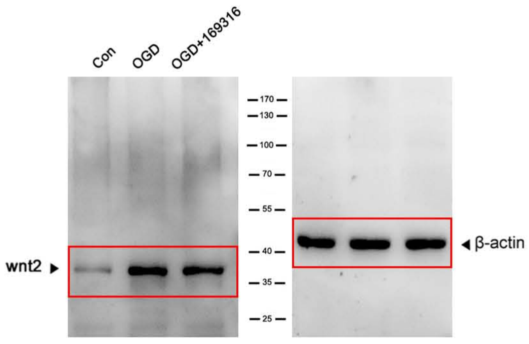


## Fig 9c

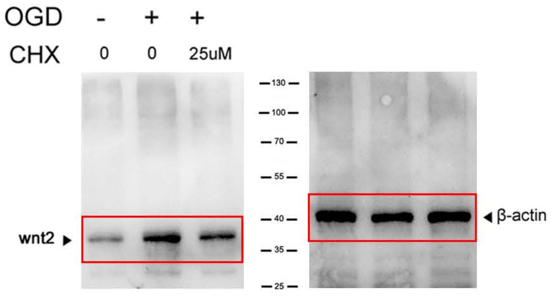


## Fig 10c

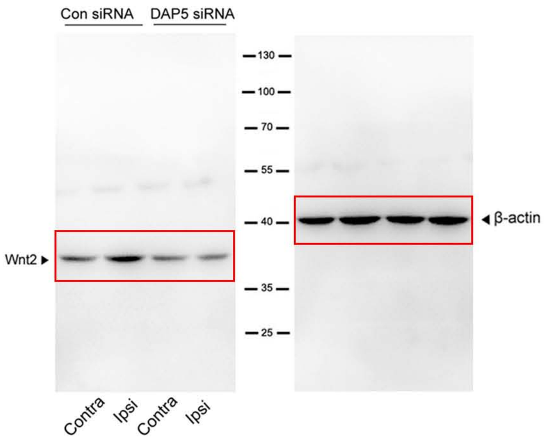

# Full gels of Supplementary Figures (biological repeat-1)

## Sup Fig 3e
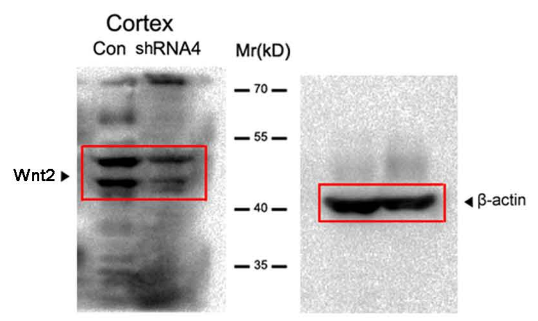


## Sup Fig 3f
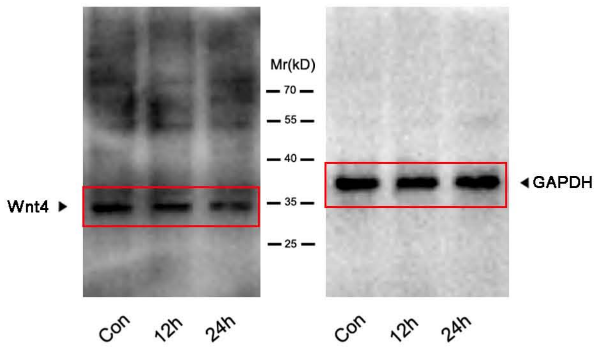


## Sup Fig 3g left
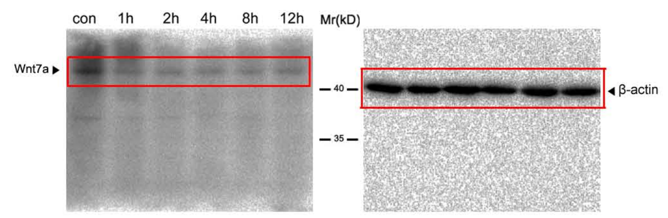


## Sup Fig 3g right
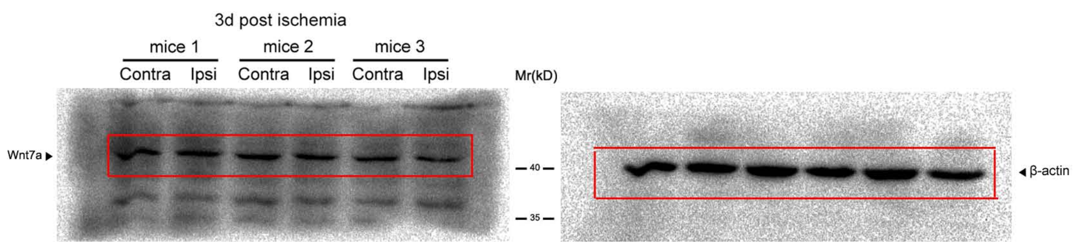


## Sup Fig 3h
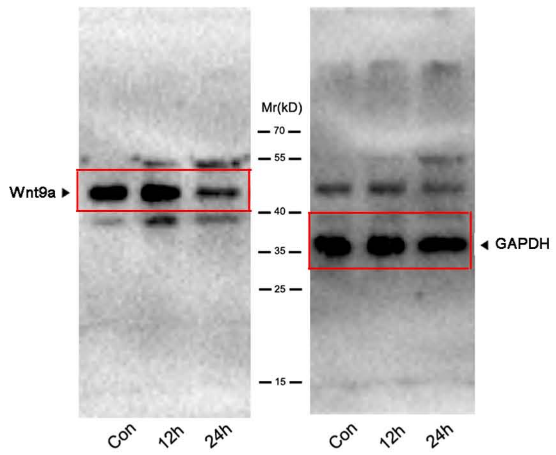


## Sup Fig 3i
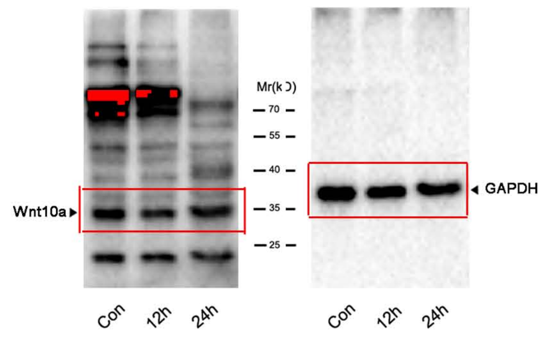


## Sup Fig 3j
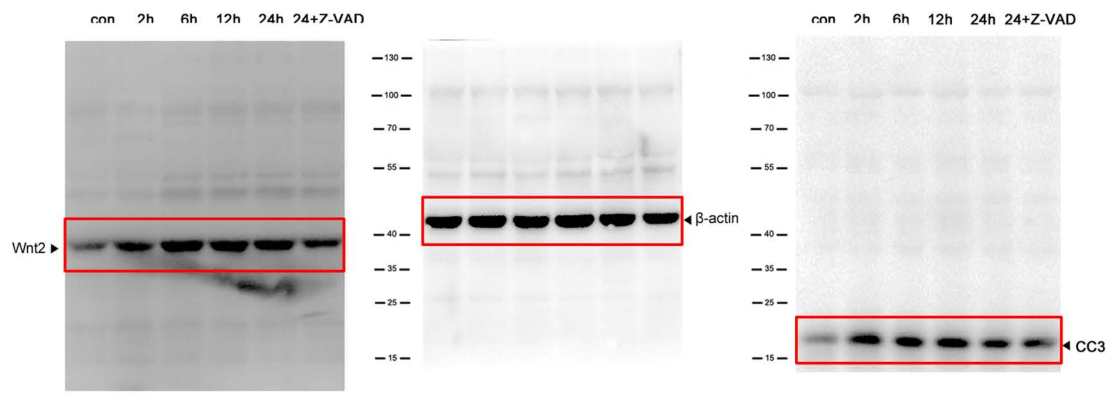


## Sup Fig 3k
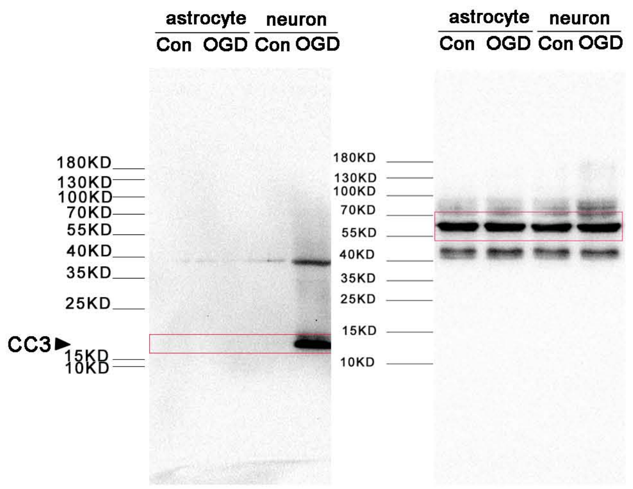


## Sup Fig 3m
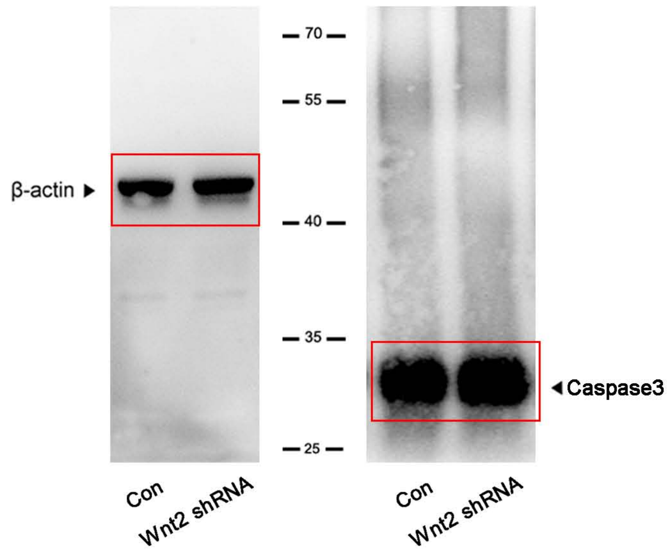


## Sup Fig 5b left
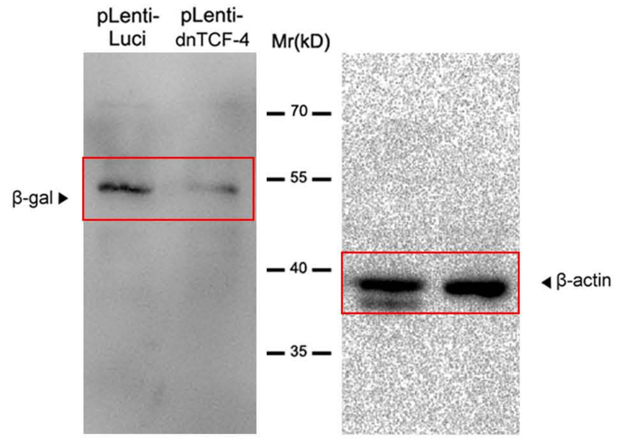


## Sup Fig 5b right
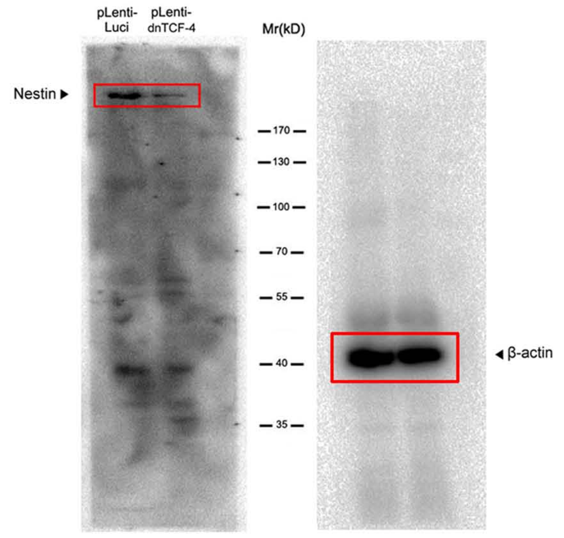


## Sup Fig 5f
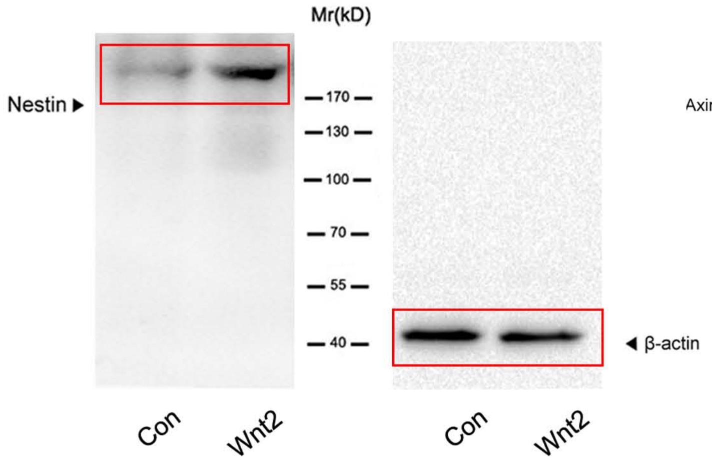


## Sup Fig 6a
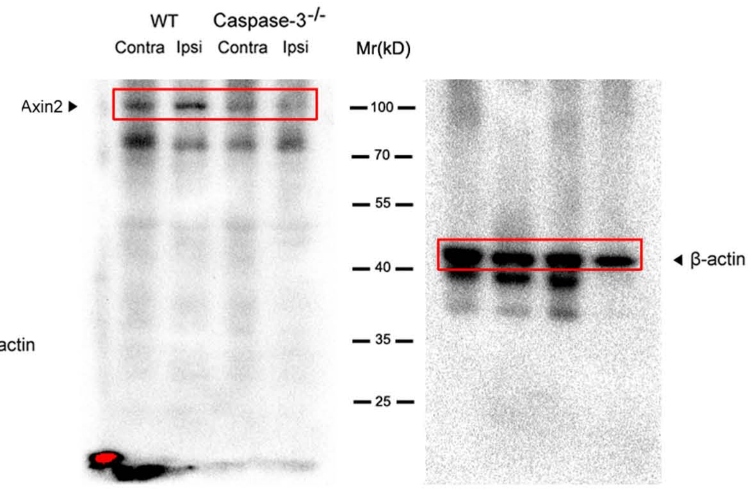


## Suo Fig 9b
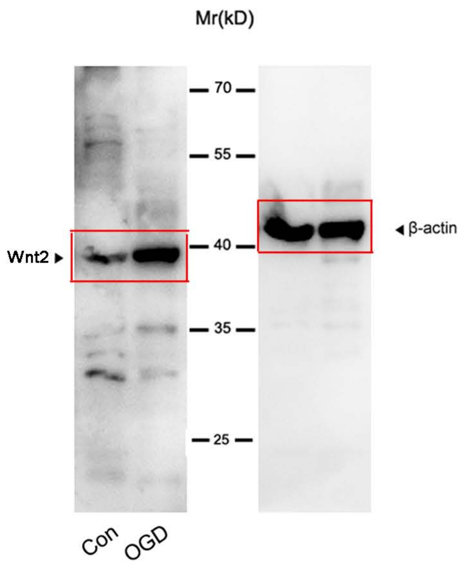


## Sup Fig 9c
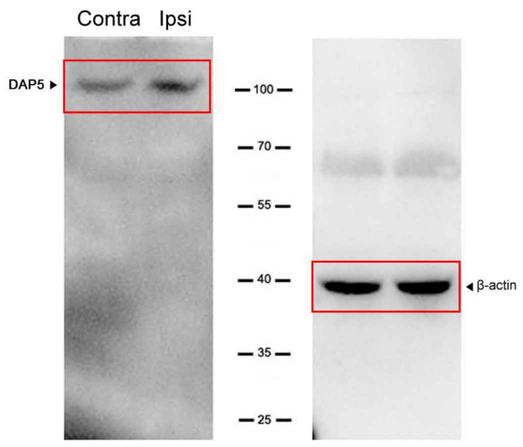


## Sup Fig 9d
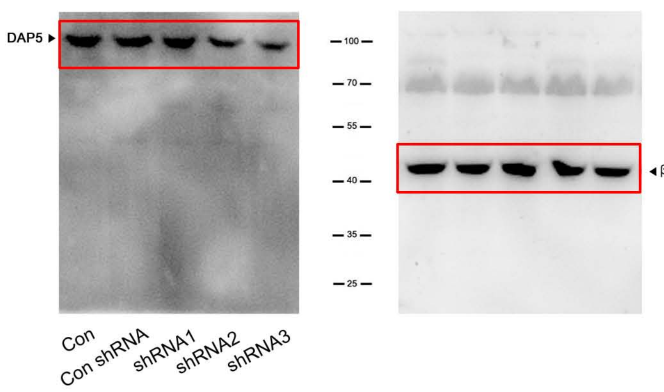


## Sup Fig 9e
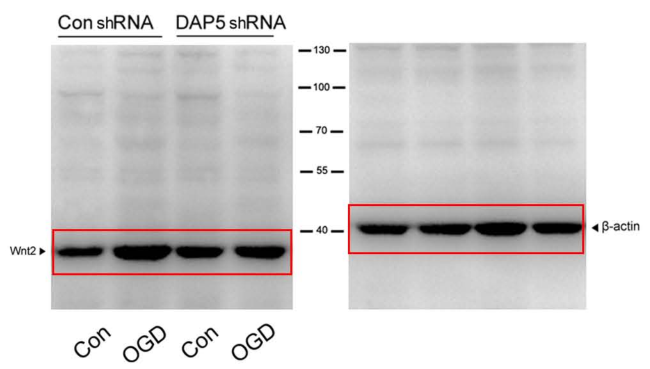

# Full gels of Supplementary Figures (biological repeat-2)

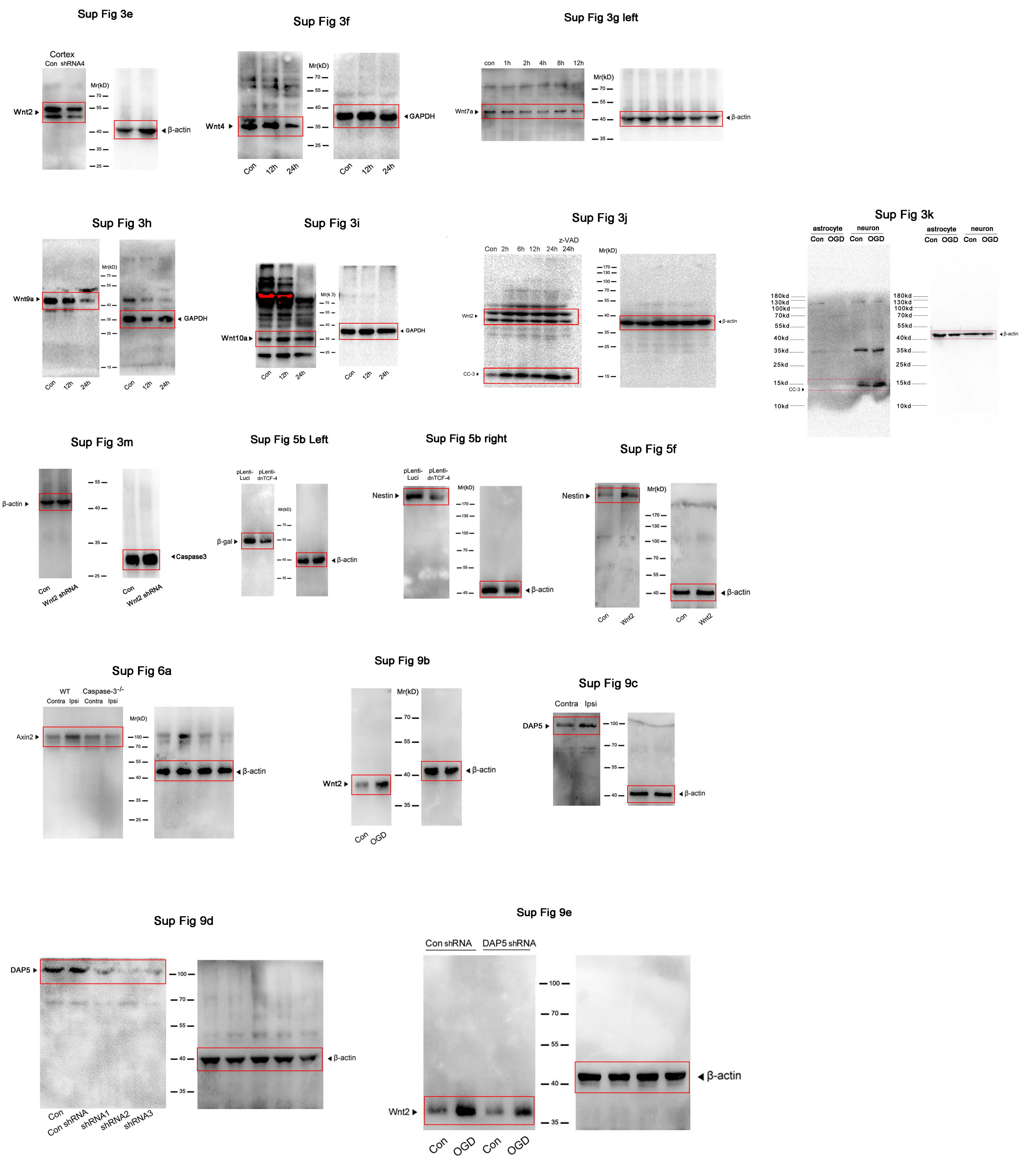

# Full gels of Supplementary Figures (biological repeat-3)

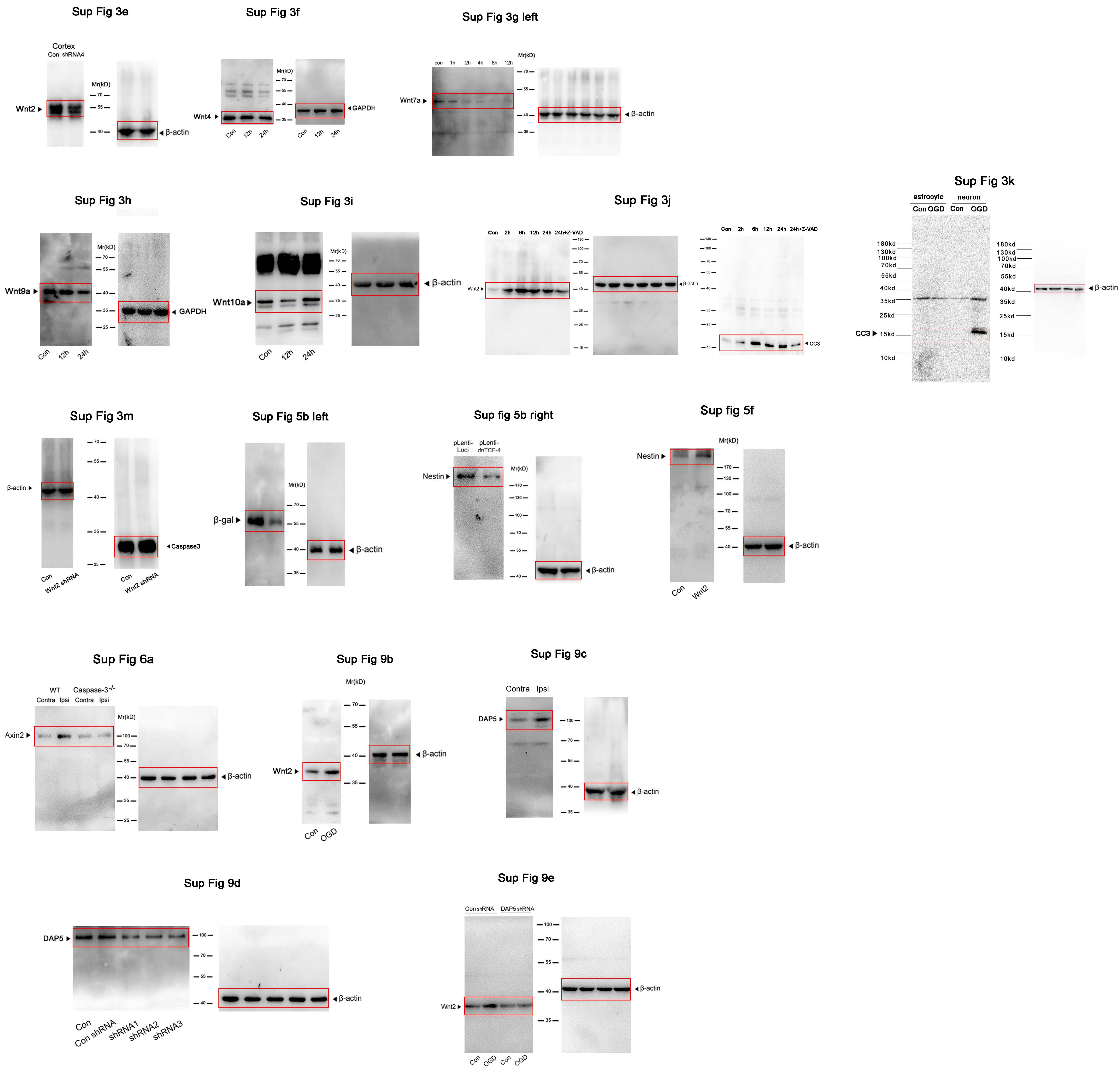

Supplement: Supplementary file 1 — Supplementary Information [file 41536_2022_248_MOESM1_ESM.pdf]
