# Supplementary material for: IRES-mediated Wnt2 translation in apoptotic neurons triggers astrocyte dedifferentiation
Source: NPJ Regen Med. 2022 Sep 2;7:42. doi: 10.1038/s41536-022-00248-1 (PMC9440034; doi:10.1038/s41536-022-00248-1)
Supplement: Supplementary file 2 — Reporting Summary [file 41536_2022_248_MOESM2_ESM.pdf]

nature portfolio

Corresponding author(s): Yazhou Wang

Last updated by author(s): Jul 17, 2022

Reporting Summary

Nature Portfolio wishes to improve the reproducibility of the work that we publish. This form provides structure for consistency and transparency in reporting. For further information on Nature Portfolio policies, see our Editorial Policies and the Editorial Policy Checklist.

Statistics

For all statistical analyses, confirm that the following items are present in the figure legend, table legend, main text, or Methods section.

n/a	Confirmed	
☐	☒	The exact sample size (*n*) for each experimental group/condition, given as a discrete number and unit of measurement
☐	☒	A statement on whether measurements were taken from distinct samples or whether the same sample was measured repeatedly
☐	☒	The statistical test(s) used AND whether they are one- or two-sided<br>*Only common tests should be described solely by name; describe more complex techniques in the Methods section.*
☒	☐	A description of all covariates tested
☐	☒	A description of any assumptions or corrections, such as tests of normality and adjustment for multiple comparisons
☐	☒	A full description of the statistical parameters including central tendency (e.g. means) or other basic estimates (e.g. regression coefficient) AND variation (e.g. standard deviation) or associated estimates of uncertainty (e.g. confidence intervals)
☐	☒	For null hypothesis testing, the test statistic (e.g. *F*, *t*, *r*) with confidence intervals, effect sizes, degrees of freedom and *P* value noted<br>*Give P values as exact values whenever suitable.*
☒	☐	For Bayesian analysis, information on the choice of priors and Markov chain Monte Carlo settings
☐	☒	For hierarchical and complex designs, identification of the appropriate level for tests and full reporting of outcomes
☒	☐	Estimates of effect sizes (e.g. Cohen's *d*, Pearson's *r*), indicating how they were calculated

Our web collection on statistics for biologists contains articles on many of the points above.

Software and code

Policy information about availability of computer code

Data collection	pCLAMP 9 was used for eletrophysiological data acquisition. Confocal images were collected with Olympus Fluoview version 3.1 in Olympus FV1000 microscope.
Data analysis	The western-blot images were quantified using ImageJ 1.50d software. Clampfit 10.6 was used for eletrophysiological data off-line analysis. GraphPad PRISM v.7.0 and SPSS 21.0 were used for statistical analysis.

For manuscripts utilizing custom algorithms or software that are central to the research but not yet described in published literature, software must be made available to editors and reviewers. We strongly encourage code deposition in a community repository (e.g. GitHub). See the Nature Portfolio guidelines for submitting code & software for further information.

Data

Policy information about availability of data

All manuscripts must include a data availability statement. This statement should provide the following information, where applicable:

- Accession codes, unique identifiers, or web links for publicly available datasets
- A description of any restrictions on data availability
- For clinical datasets or third party data, please ensure that the statement adheres to our policy

The authors declare that all data supporting the findings of this study are available within the paper and the supplementary information.

Field-specific reporting

Please select the one below that is the best fit for your research. If you are not sure, read the appropriate sections before making your selection.

☒ Life sciences ☐ Behavioural & social sciences ☐ Ecological, evolutionary & environmental sciences

For a reference copy of the document with all sections, see nature.com/documents/nr-reporting-summary-flat.pdf

Life sciences study design

All studies must disclose on these points even when the disclosure is negative.

Sample size	Sample size for each experiment is clearly indicated in the figure legends for each experiment. The sample size was chosen based on previous experience. No statistical methods were used to predetermine sample size.
Data exclusions	For electrophysiological recordings, neurons were excluded when the resting membrane potential were positive than -55 mV and action potentials did not have overshoot or the series resistance changed by >20%.
Replication	Experimental replication was performed as stated in the manuscript text.
Randomization	Mice were randomly assigned to control and treatment group.
Blinding	Experimenter was blinded to the identity of mice being analyzed in behavioral tests, electrophysiological recording and immunohistochemical staining experiments.

Reporting for specific materials, systems and methods

We require information from authors about some types of materials, experimental systems and methods used in many studies. Here, indicate whether each material, system or method listed is relevant to your study. If you are not sure if a list item applies to your research, read the appropriate section before selecting a response.

Materials & experimental systems

n/a	Involved in the study	
☐	☒	Antibodies
☒	☐	Eukaryotic cell lines
☒	☐	Palaeontology and archaeology
☐	☒	Animals and other organisms
☒	☐	Human research participants
☒	☐	Clinical data
☒	☐	Dual use research of concern

Methods

n/a	Involved in the study	
☒	☐	ChIP-seq
☒	☐	Flow cytometry
☒	☐	MRI-based neuroimaging

Antibodies

Antibodies used	See supplementary information.
Validation	Validation was made by the manufacturers.

Animals and other organisms

Policy information about studies involving animals; ARRIVE guidelines recommended for reporting animal research

Laboratory animals	The Topgal mice (Jax stock No. 004623), caspase-3 knockout mice (Jax stock No. 006233) and ROSA-DTA mice (Jax stock No. 009669)were obtained from Jackson lab. Nestin-CreER mice were gifted from Dr. Ryoichiro Kageyama (Kyoto University). Wild type C57 mice were obtained from the Fourth Military Medical University. Macaca Mulatta were obtained from Kunming Institute of Zoology, Chinese Academy of Sciences.
Wild animals	N/A
Field-collected samples	N/A
Ethics oversight	All mice experiments were carried out under protocols approved by the Animal Care and Use Committees of Fourth Military Medical University. All the monkey experiments were performed under the approval of the Animal Care and Use Committees of Kunming Institute of Zoology, Chinese Academy of Sciences (IACUC19009).

Note that full information on the approval of the study protocol must also be provided in the manuscript.
